# Nanoscale Hybrid Amorphous/Graphitic Carbon as Key Towards Next‐Generation Carbon‐Based Oxidative Dehydrogenation Catalysts

**DOI:** 10.1002/anie.202014862

**Published:** 2021-01-26

**Authors:** Felix Herold, Stefan Prosch, Niklas Oefner, Kai Brunnengräber, Oliver Leubner, Yannick Hermans, Kathrin Hofmann, Alfons Drochner, Jan P. Hofmann, Wei Qi, Bastian J. M. Etzold

**Affiliations:** ^1^ Department of Chemistry Ernst-Berl-Institut für Technische und Makromolekulare Chemie Technical University of Darmstadt Alarich-Weiss-Strasse 8 64287 Darmstadt Germany; ^2^ Department of Materials and Earth Sciences Surface Science Laboratory Technical University of Darmstadt Otto-Berndt-Strasse 3 64287 Darmstadt Germany; ^3^ Department of Chemistry Eduard-Zintl-Institut für Anorganische und Physikalische Chemie Technical University of Darmstadt Alarich-Weiss-Strasse 12 64287 Darmstadt Germany; ^4^ Shenyang National Laboratory for Material Science Institute of Metal Research Chinese Academy of Sciences Wenhua Road 72 Shenyang 110016 People's Republic of China

**Keywords:** carbon materials, heterogeneous catalysis, oxidative dehydrogenation

## Abstract

A new strategy affords “non‐nano” carbon materials as dehydrogenation catalysts that perform similarly to nanocarbons. Polymer‐based carbon precursors that combine a soft‐template approach with ion adsorption and catalytic graphitization are key to this synthesis strategy, thus offering control over macroscopic shape, texture, and crystallinity and resulting in a hybrid amorphous/graphitic carbon after pyrolysis. From this intermediate the active carbon catalyst is prepared by removing the amorphous parts of the hybrid carbon materials via selective oxidation. The oxidative dehydrogenation of ethanol was chosen as test reaction, which shows that fine‐tuning the synthesis of the new carbon catalysts allows to obtain a catalytic material with an attractive high selectivity (82 %) similar to a carbon nanotube reference, while achieving 10 times higher space–time yields at 330 °C. This new class of carbon materials is accessible via a technically scalable, reproducible synthetic pathway and exhibits spherical particles with diameters around 100 μm, allowing unproblematic handling similar to classic non‐nano catalysts.

## Graphical abstract

An amorphous/graphitic hybrid material is synthesized by growing nanoscale graphite crystallites in a non‐nano polymer‐derived carbon by catalytic graphitization. An active dehydrogenation catalyst is obtained by creating accessibility to these graphitic domains via selective oxidation. This carbon dehydrogenation catalyst offers a 10‐fold increase in space‐time‐yield in the oxidative dehydrogenation of ethanol compared to a carbon nanotube benchmark.

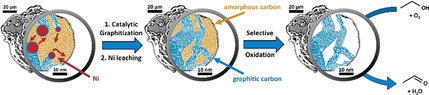

## Introduction

Catalysts are key materials in modern society that enable selective transformation of raw materials into valuable products while avoiding waste and saving energy.^[1]^ In case of industrially relevant oxidative dehydrogenation reactions (ODH), most known catalyst systems are based on transition metals (e.g. Fe, V, Mo, Ag).^[2]^ Due to the drawbacks associated with the use of transition metals, such as rare occurrence, environmentally harmful mining procedures and toxicity, it is highly interesting that pure carbon was shown to exhibit catalytic activity in these types of reactions and could be a sustainable substitution.[^3^, ^4^] Typical reactions reported with carbon catalysts are the gas‐phase oxidative dehydrogenations of ethane,^[5]^ propane,^[6]^ butane,^[7]^ and ethylbenzene,^[8]^ which are dehydrogenated to the corresponding olefins. Beyond alkanes, alcohols like 1‐propanol and ethanol have been oxidized to the corresponding aldehydes using carbon catalysts.^[9]^

Up to date, the development of carbon‐based catalysts for oxidative dehydrogenation reactions may be divided into two generations. First‐generation carbon catalysts were inspired by the discovery of the catalytic activity of coke deposits on metal‐based catalysts for the oxidative dehydrogenation of ethylbenzene.^[3]^ In this context, mainly amorphous carbonaceous materials, such as activated carbon and carbon black, were investigated.[^10^, ^11^] Despite showing significant activity and selectivity, these early catalysts suffered from inadequate oxidation stability and were subsequently succeeded by the second generation of carbon‐based dehydrogenation catalysts, represented by carbon nanomaterials.[^11^, ^12^] A wide variety of carbon nanomaterials, for example, carbon nanotubes,^[11]^ carbon nanofibers,^[13]^ onion‐like carbon,^[14]^ and few‐layered graphene,^[15]^ have been employed successfully in oxidative dehydrogenation reactions. The benefit of carbon nanomaterials compared to amorphous first generation catalysts is mainly their crystalline, predominantly sp^2^‐hybridized microstructure that is responsible for sufficient oxidation resistance and simultaneously enables high redox activities.[^16^, ^17^] In this context, the presence of large conjugated (graphitic) domains with a high density of defect sites (e.g. edges, in‐plane defects) seems to be fundamental. These structures enable high redox activity by acting as electron storage for conjugated oxygen surface groups, such as ketonic carbonyl groups, anchored at edges and defects.[^13^, ^15^, ^17^, ^18^, ^19^] As no inner porosity is present in carbon nanomaterials, these active centers are situated at the outer surface and are therefore highly accessible.

However, in case of heterogeneous catalysis, due to intrinsic drawbacks such as the large pressure drop and high porosity of fixed nanocarbon catalyst beds, a demanding scaling of synthesis and unclear health hazards, nanocarbon materials are still awaiting industrial application.[^20^, ^21^]

**Scheme 1:**
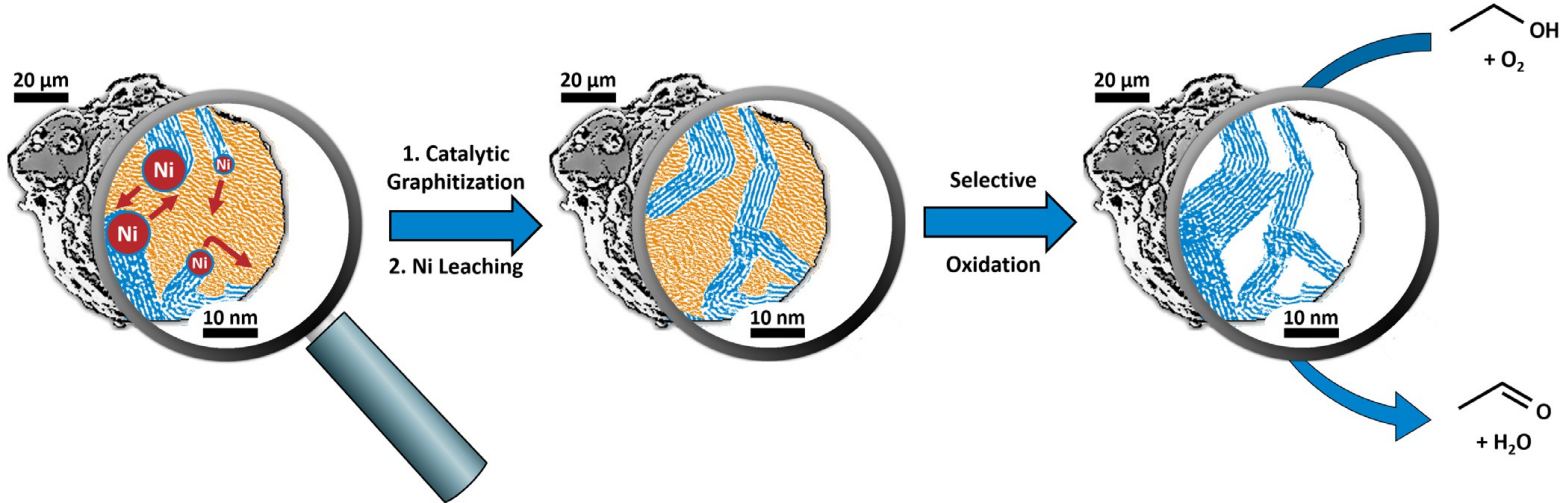
A hybrid amorphous/graphitic material is prepared by catalytic graphitization of a polymer‐based carbon precursor. Removal of amorphous/turbostratic domains yields the active dehydrogenation catalyst by providing access to defect‐rich graphitic domains.

Initial studies with carbide‐derived carbons showed that highly crystalline but mesoporous carbon powders seem to exhibit the needed key catalytic features and show similar catalytic properties as carbon nanomaterials.^[21]^ Nevertheless, carbide‐derived carbons are currently research model materials, as the necessary chlorination of carbides at temperatures above 1200 °C hinders application as a technical catalyst. Inspired by these results, a new generation of carbon‐based dehydrogenation catalysts is introduced here, where the key features for high activity and stability are added to conventional powder carbons by employing a simple and scalable polymer‐based synthetic pathway. Besides scalability, polymers have the advantage that the synthesis is very reproducible, while the use of purified monomers enables the synthesis of highly defined carbon precursors that contain a minimum of impurities. As nano‐sized graphite domains are anchoring points for active sites but should be embedded in macroscopic particles, the approach of this work is to grow crystallites within the carbon matrix by catalytic graphitization during pyrolysis of the polymer precursor. As a consequence of this approach, carbon crystallization only occurs in domains which come into contact with the graphitization catalyst, and a hybrid carbon material, consisting of amorphous and graphitic domains, results. Finally, the active oxidative dehydrogenation catalyst is obtained by creating access to these graphitic domains via selective oxidation of the amorphous parts of the material (Scheme 1).

Key to a successful catalyst synthesis following Scheme 1 is the amorphous/graphitic hybrid carbon with heterogeneity in terms of crystallinity at the nanoscale. To obtain such materials, finely dispersed metal particles (e.g. Ni, Co, Fe) are a prerequisite and can be obtained through a homogeneous distribution of metal ions within the precursor polymer.[^22^, ^23^, ^24^] Such homogeneous distributions can be realized by employing a polymer precursor with ion‐exchange properties, which makes up the first requirement for the polymer precursor. As macroscopic powders shall result, the second requirement is good accessibility of the carbon resulting from pyrolysis. Therefore, a suitable template should be incorporated into the precursor polymer to ensure mesoporosity of the carbon material after pyrolysis.

**Scheme 2:**
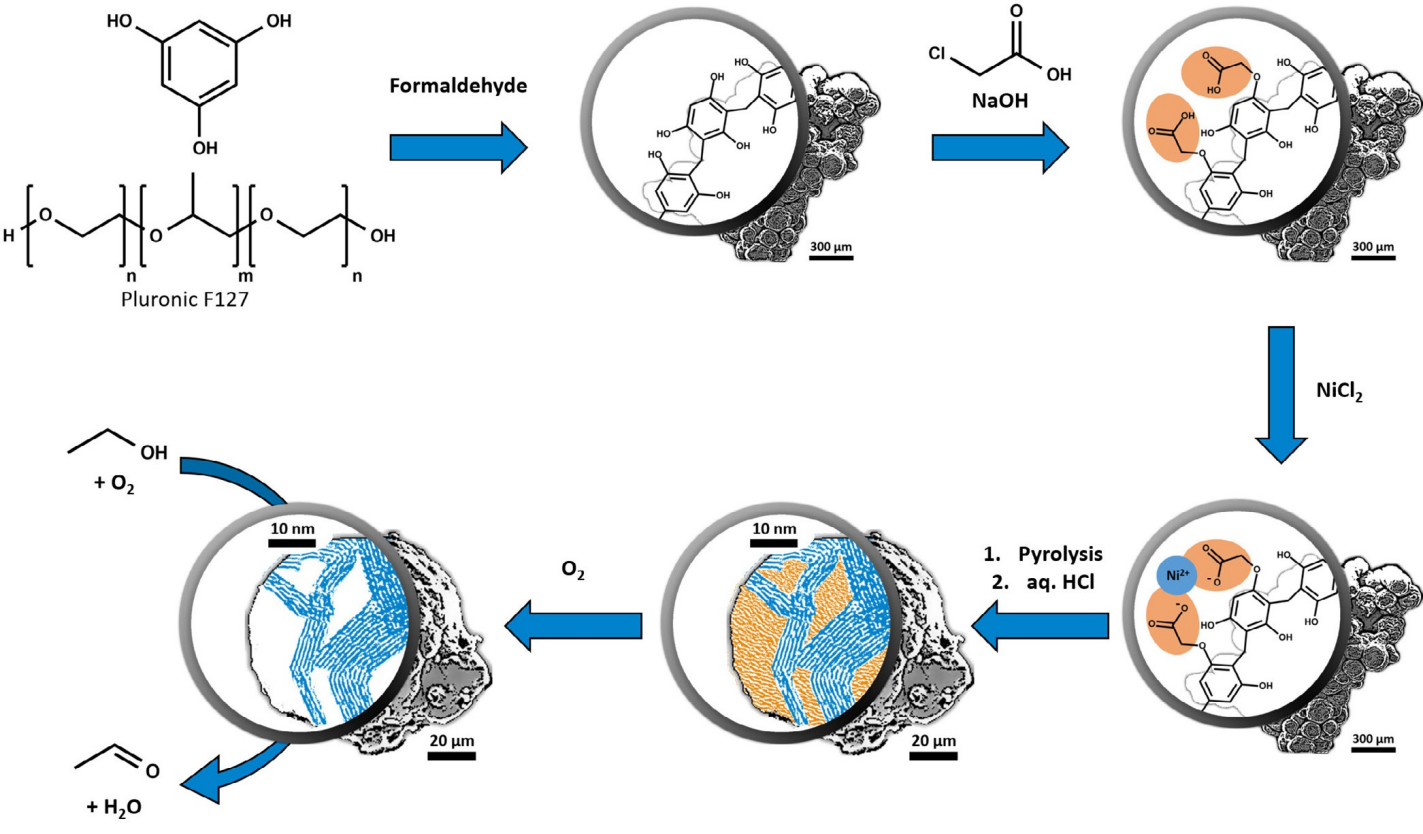
A hybrid amorphous/graphitic material is prepared by catalytic graphitization of a polymer‐based carbon precursor. Removal of amorphous/turbostratic domains yields the active dehydrogenation catalyst by providing access to defect‐rich graphitic domains.

Based on these prerequisites for the new synthesis route, the polycondensation in solution of a phloroglucinol/formaldehyde reaction system in presence of the soft template Pluronic F127 is chosen to predetermine morphology and ensure a mesoporous texture (Scheme 2).^[25]^ To add ion exchange capacity, the precursor polymer is modified with carboxylic acid groups anchored on the polymer surface. This precursor can subsequently be loaded with metal ions via ion exchange, resulting in a homogeneous, atomic distribution. Pyrolysis of this carbon precursor initially leads to carbonaceous material and then to finely dispersed graphitization catalyst particles through carbothermal reduction of the metal ions. At elevated temperatures (>700 °C), these metal particles migrate through the carbon matrix and graphitize surrounding domains by forming metastable metal carbides, which subsequently decompose to form graphitic carbon.[^22^, ^23^] In this manner, a hybrid material with heterogeneity at the nanoscale is generated: domains which did not come into contact with the graphitization catalyst exhibit an amorphous/turbostratic microstructure, while domains that came into contact with the graphitization catalyst are highly crystalline.

Edges and defects of graphite crystallites grown by this approach are initially buried in a matrix of amorphous/turbostratic carbon.

The difference in oxidation stability between graphitic and amorphous/turbostratic carbon can now be exploited to create access to these structural key features by selective oxidation of non‐graphitic domains.

Within this contribution this synthesis route towards novel carbon‐based dehydrogenation catalysts is studied and the new catalytic materials are tested for their catalytic performance for the oxidative dehydrogenation of ethanol to acetaldehyde. This reaction is of great practical interest as it represents a catalytic link between bioethanol, which is easily obtainable from renewable resources, and an important intermediate in current industrial chemistry.^[26]^

## Results and Discussion

A monomer system of phloroglucinol (1,3,5‐trihydroxy benzene) and formaldehyde that was subjected to polycondensation in presence of the triblock copolymer soft template Pluronic F127 (according to a procedure of Chai et al.^[25]^) served as starting material for the catalyst synthesis. This approach yields spherical polymer particles of about 250 μm diameter in which micelles of the triblock copolymer Pluronic F127 are incorporated in a cross‐linked matrix of phloroglucinol (subsequently denoted as precursor polymer, Figure S1). Aiming to introduce negatively charged groups to the surface of the precursor polymer, ubiquitous acidic phenolic hydroxy groups were subjected to etherification with chloroacetic acid (subsequently denoted as carboxy polymer). This surface manipulation only had a minor impact on the pyrolysis behavior compared to the precursor polymer, as indicated by thermogravimetric analysis (Figure S2), while a significantly enhanced ion exchange capacity (from 0.89 to 2.44 mmol g^−1^) was proven by potentiometric titration (Figure S3). Analysis of the carboxy polymer by diffuse reflectance infrared Fourier transform spectroscopy (DRIFTS) revealed a band at 1760 cm^−1^ that can be assigned to individual (non‐dimer) carboxylic acid groups which could neither be detected in the spectra of the precursor polymer nor in the spectra of the main constituents of the polymers (Figure S4). By exchanging H^+^ for Ni^2+^, a nickel loading for the polymer of 1.41±0.26 wt % and 1.15±0.07 wt % could be determined by temperature programmed oxidation (TPO) and inductive coupled plasma optical emission spectroscopy (ICP‐OES), respectively (Figure S5).

**Figure 1:**
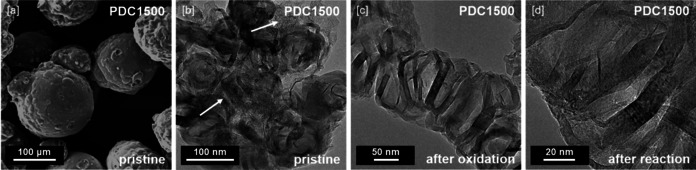
[a] SEM picture of PDC1500‐P. [b] TEM image of pristine hybrid amorphous/graphitic PDC1500 (PDC1500‐P, white arrows mark amorphous/turbostratic domains, graphitic domains can be identified by darker, ribbon‐like structures). [c] TEM images of PDC1500 after the removal of amorphous/turbostratic carbon by oxidation (PDC1500‐AO) and [d] of PDC1500 after utilization as catalyst in the oxidative dehydrogenation of EtOH (PDC1500‐AR).

**Figure 2:**
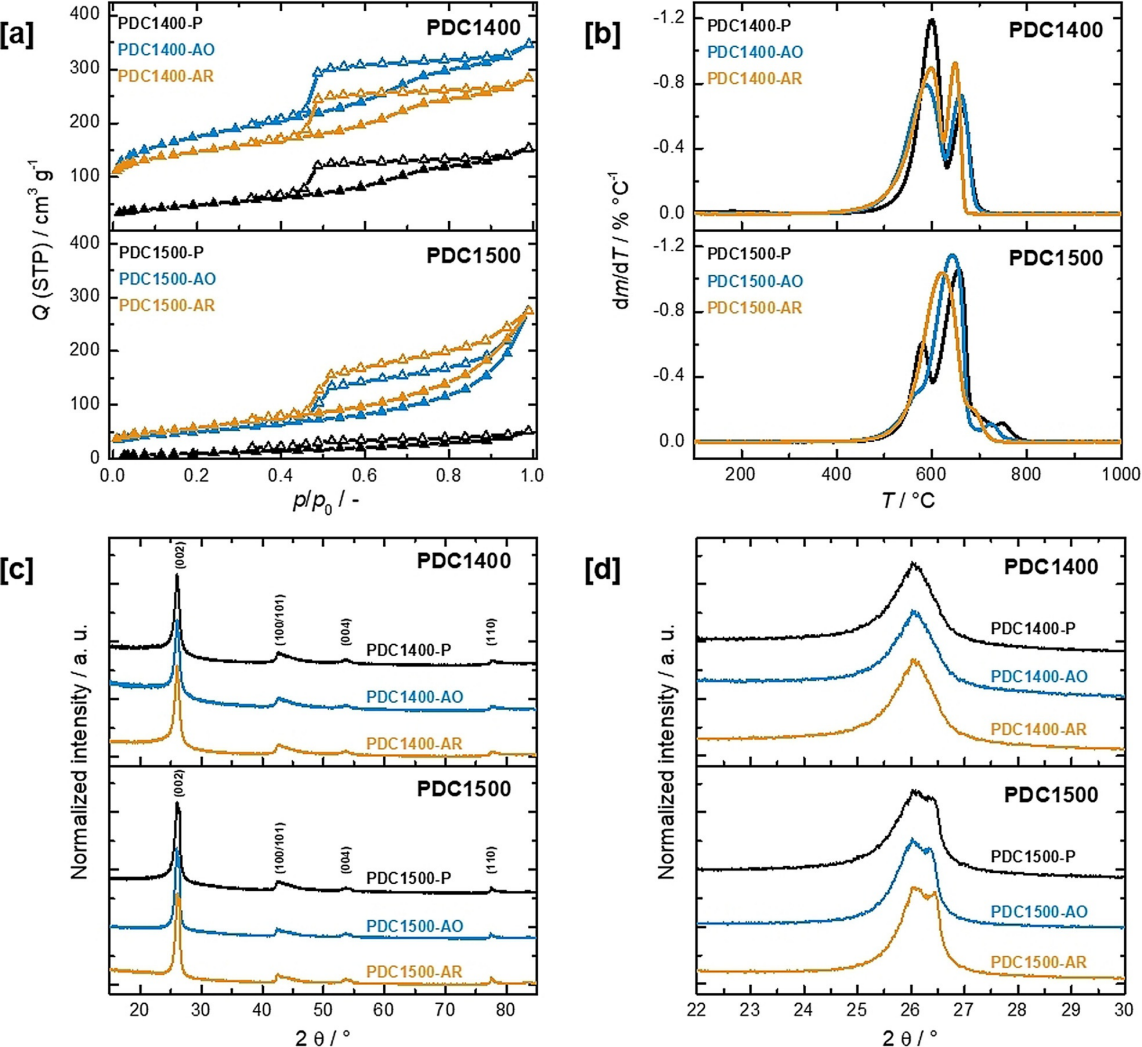
[a] N_2_‐physisorption isotherms of polymer‐derived carbons, [b] temperature programmed oxidation in air of polymer derived carbons, [c] X‐ray powder diffraction patterns of polymer derived carbons, [d] detail of the (002) reflection in X‐ray diffractograms of PDC1400 and PDC1500.

Pyrolysis of Ni‐loaded polymer particles at 1400 and 1500 °C yields spherical carbon particles of about 100 μm diameter (note: after HCl washing, pristine polymer‐derived carbons are denoted as PDCXXXX‐P, where XXXX represents the pyrolysis temperature; Figure 1 a). The textural properties of the pristine PDC1400‐P and PDC1500‐P materials were characterized using N_2_ physisorption (Figure 2 a). The isotherms show type IV(a) behavior, indicating the presence of meso‐ and micropores. The hysteresis loops of the isotherms exhibit a steep desorption branch in the relative pressure range of 0.4<*p*/*p*_0_<0.6 and can be classified as type H2(a).^[27]^ Specific surface areas were determined to be 171 m^2^ g^−1^ and 38 m^2^ g^−1^ for PDC1400‐P and PDC1500‐P, respectively. Raman spectroscopy of PDC1400‐P and PDC1500‐P shows distinct first‐order D‐ and G‐Bands at 1350 cm^−1^ and 1580 cm^−1^, respectively, as well as clearly developed second‐order D‐Bands at 2700 cm^−1^, indicating a defect‐rich graphitic carbon material (Figure S6).^[22]^ Compared to PDC1500‐P, PDC1400‐P shows a higher average *I*_D_/*I*_G_ ratio (obtained from first‐order spectra; 0.51 and 1.05, respectively). Furthermore, the distribution of *I*_D_/*I*_G_ values derived from 10 Raman spectra of a given sample is significantly broader in case of PDC1400‐P, indicating a higher degree of inhomogeneity in terms of crystallinity (Figure S7–S9). TPO analysis shows the occurrence of two carbon species with different oxidation resistances for both PDC1400‐P and PDC1500‐P (Figure 2 b). The mass fraction of the carbon species with low oxidation resistance (LOR) and high oxidation resistance (HOR) can be estimated from the residual mass at the infliction point of the TPO mass loss curve (Figure S10). For PDC1400‐P, the amount of species with LOR is significantly higher (LOR/HOR 71:29), while for PDC1500‐P this ratio is reversed and the carbon species with higher oxidation resistance dominates (LOR/HOR 31:69). After thorough washing with HCl, no Ni residue could be detected by TPO. It should be noted at this point that other techniques such as X‐ray powder diffraction (XRD) transmission electron microscopy (TEM) as well as ICP‐OES analysis failed to provide evidence of relevant Ni residues (Table S1).

XRD analysis of PDC1400‐P and PDC1500‐P catalytically graphitized carbons reveals pronounced reflections that can be assigned to the graphite lattice planes (002), (100/101), (004), and (110) (Figure 2 c). Compared to crystalline graphite‐2 H, the reflections of PDC1400‐P are shifted to lower 2*θ* values and exhibit broad, to some extent asymmetric profiles characteristic for graphitic phases with a high degree of disorder.[^23^, ^28^] In contrast to the powder diffraction pattern of PDC1400‐P, the (002) and (004) reflections of PDC1500‐P exhibit pronounced shoulders at the higher 2*θ* regions of these peaks, suggesting superposition of two reflections (Figure 2 c,d and Figure S11 showing also graphite‐2 H). Deconvolution of the data results in two peaks located at 26.17° and 26.50°/2*θ* for the (002) and at 53.74° and 54.54°/2*θ* for the (004) reflection. A similar peak splitting was recently described by Alaferdov et al. for sonicated graphite samples and attributed to graphitic phases exhibiting different degrees of order.^[29]^ The results of XRD and Raman spectroscopy point to the coexistence of two graphitic phases: one highly disordered and another exhibiting a higher degree of order.^[29]^ It needs to be noted that polymer samples without Ni loading but pyrolyzed at identical temperatures exhibit merely broad reflections and a low signal‐to‐noise ratio, suggesting an X‐ray amorphous character and demonstrating the importance of the graphitization catalyst to obtain a carbon material with a nanoscale hybrid amorphous/graphitic microstructure (Figure S12).

Supporting results of previous analyses, TEM reveals domains of extended graphitic structures and large amorphous/turbostratic parts for both PDC1400‐P and PDC1500‐P. At the mesoscopic scale, these crystalline/amorphous hybrid materials show graphitic domains exhibiting a large degree of disorder. At the microscopic scale, the catalytic graphitization with Ni seems to produce a rather imperfect form of graphite, as various forms of defects, such as buckling, merging and splitting of graphene layers, can be observed in the graphitized parts of PDC1400‐P and PDC1500‐P (Figure 1 b and Figures S13–S14).

Oxidation of the pristine PDC materials in synthetic air at 380 °C did not lead to an observable degradation of the macrostructure (Figure S15) but induced distinct textural changes (denoted as ‘after oxidation’, PDCXXXX‐AO). In case of PDC1400‐AO, a significant rise in N_2_ uptake in the low relative pressure range 0<*p*/*p*_0_<0.05 could be detected, indicating increased microporosity (Figure 2 a). Simultaneously, the specific surface area increases from 171 m^2^ g^−1^ for PDC1400‐P to 640 m^2^ g^−1^ for PDC1400‐AO. To a certain extent, this also applies for PDC1500‐AO but the most prominent change in the isotherm compared to the pristine material is the rise in N_2_ uptake at high relative pressures 0.5<*p*/*p*_0_<1. No plateau in N_2_ uptake at high relative pressures can be detected and the desorption hysteresis changes from type H2(a) to H4. The described changes of the N_2_ isotherm indicate a slight increase in microporosity for PDC1500‐AO, and the development of pronounced mesoporosity.^[27]^ In case of PDC1500, oxidative treatment increases the specific surface area from 38 m^2^ g^−1^ to 182 m^2^ g^−1^. In comparison to the pristine materials, Raman spectroscopy reveals a decrease in the average *I*_D_/*I*_G_ ratio for PDC1400‐AO (1.05 to 0.91) and PDC1500‐AO (0.51 to 0.39) (Figure S6). In addition, a narrowing distribution of the *I*_D_/*I*_G_ values over 10 spectra could be observed for both materials. These findings indicate that oxidized PDC possesses a higher ordered microstructure than pristine materials, while the inhomogeneity of the material appears to decrease with oxidative treatment (Figures S8 and S9). TPO measurements show a shift in the ratio between oxidation‐stable and ‐unstable carbon species: the mass fraction of the less oxidation‐resistant carbon species decreases during oxidation of the pristine PDC materials (Figure 2 b). Despite removing some of the carbon material of low oxidation resistance of PDC1400‐P, PDC1400‐AO still contains significant amounts thereof (LOR/HOR 67:33). In contrast, oxidative treatment removed the greatest part of the less oxidation‐resistant species for PDC1500‐AO (LOR/HOR 15:85). XRD suggests that the oxidation procedure did not provoke any decline in crystallinity, as position and shape of the observed reflexes did not change significantly for PDC1400‐AO or PDC1500‐AO (Figure 2 c,d). TEM analysis of PDC1500‐AO showed a loss of amorphous/turbostratic domains after oxidation in synthetic air. While the graphitized parts of the material did not seem to be affected by the oxidation, most of the amorphous matrix appears to be eliminated (Figure 1 c, Figure S14).

**Figure 3:**
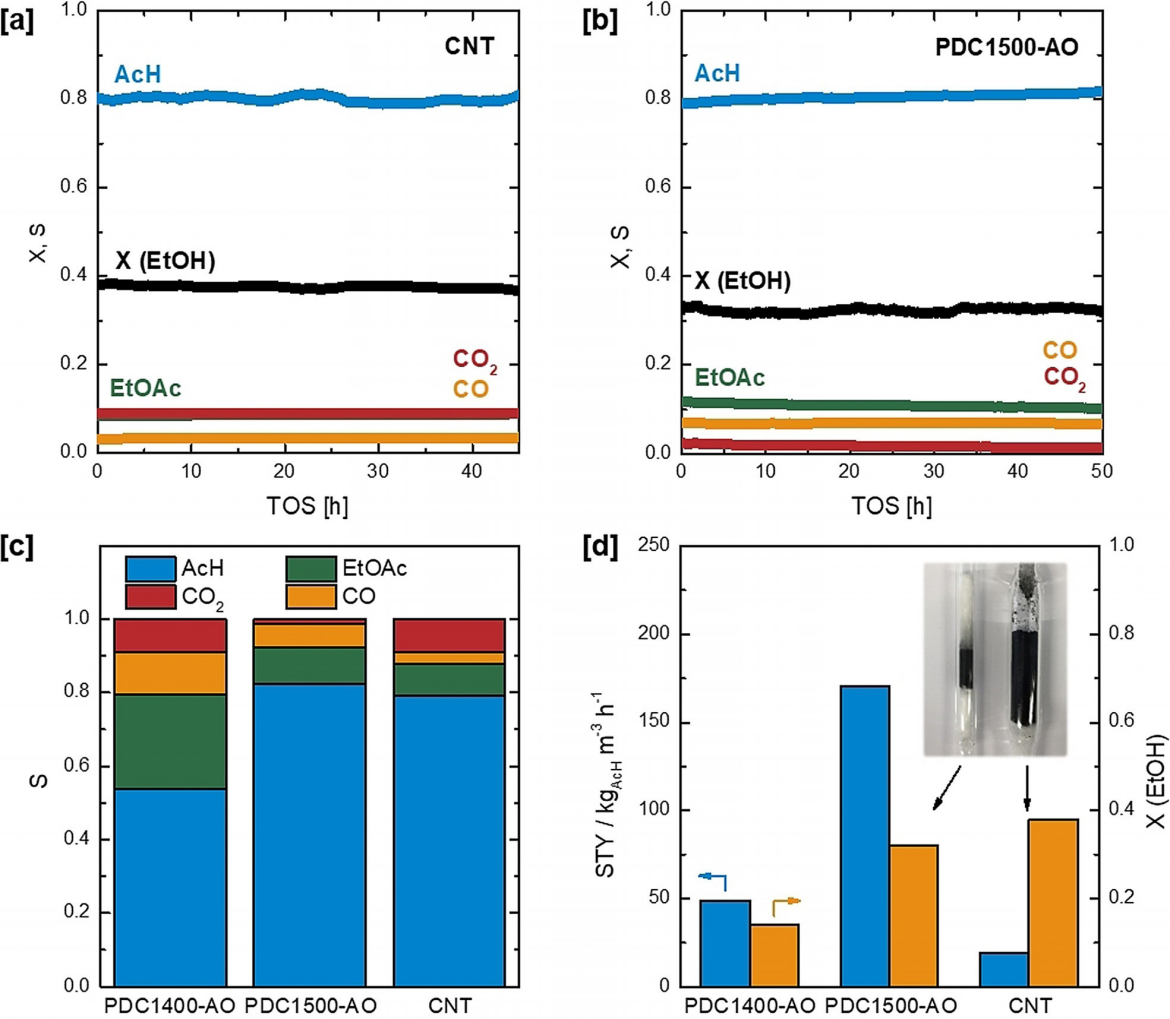
[a] Conversion of ethanol on the CNT benchmark catalyst over 45 h TOS. [b] Conversion of ethanol on PDC1500‐AO over 50 h TOS. [c] Selectivity towards acetaldehyde, ethyl acetate, CO, and CO_2_ for PDC1400‐AO, PDC1500‐AO, and the CNT benchmark catalyst. [d] Space–time yield of acetaldehyde and conversion for PDC1400‐AO, PDC1500‐AO, and CNT catalysts and pictures of the fixed catalyst beds of PDC1500‐AO and CNT (see Figure S19). Catalytic testing was conducted using 90 mg of catalyst at 330 °C in a tubular fixed bed reactor with 4.3 vol % EtOH, 10 vol % O_2_ at a total volume flow of 20 mL min^−1^ (STP) with He as balance.

After thorough characterization of the new generation of carbonaceous materials, the gas‐phase oxidation of ethanol (EtOH) to acetaldehyde (AcH) at 330 °C is employed as a test reaction to assess the catalytic activity of the new PDC materials. Multi‐walled carbon nanotubes (CNTs) as typical 2^nd^ generation catalysts serve as benchmark (Figures 3 and S16–S17, studies were exclusively carried out within the regime of constant reaction rates under exclusion of film diffusion limitations (see side note S1)). Besides acetaldehyde, ethyl acetate (EtOAc) as well as carbon monoxide (CO) and carbon dioxide (CO_2_) were detected as side products for the CNT catalyst. A selectivity towards AcH of 79 % was obtained (at an EtOH conversion of 38 %), whereas the selectivity to the side products EtOAc, CO, and CO_2_ amounted to 9 %, 3 %, and 9 %, respectively. Interestingly, at similar conversion PDC1500‐AO also shows an excellent selectivity towards AcH of 82 % (Figure 3 b,c). In contrast, the resulting EtOH conversion and selectivity of PDC1400‐AO remains inferior compared to the CNT benchmark. At the expense of selectivity towards AcH (54 %), PDC1400‐AO displays a high selectivity towards the side products EtOAc (26 %), CO (11 %), and CO_2_ (9 %) (Figures 3 c,d and S18).

Regarding activity comparison, the technically relevant volume‐based space–time yield is employed. In this context, the great advantage of the new generation non‐nanocarbon catalysts compared to the carbon nanotube benchmark is revealed. Due to the pronounced higher catalyst bed density of the PDC catalysts compared to the CNTs, the space–time yield of acetaldehyde at 330 °C was found to be nearly one order of magnitude higher for PDC1500‐AO (171 kg m^−3^ h^−1^) compared to CNTs (19 kg m^−3^ h^−1^) (Figures 3 d, S19). For PDC1400‐AO the production rate is lower in comparison to PDC1500‐AO by a factor of 3.5.

In addition to the superior activity of PDC1500‐AO, the new materials must also exhibit the high stability that is characteristic for 2^nd^ generation nano‐carbon catalysts. An experiment with 50 h on stream (carbon balance at 99.70±3 %), without observable loss of activity and change in selectivity was carried out and highlights the stability of the new generation PDC1500‐AO catalyst (Figure 3 b). SEM analysis of the used PDC catalysts (denoted as ‘after reaction’, PDCXXXX‐AR) showed that even extended periods (>50 h) on stream did not induce observable degradation of the macrostructure (Figures S15). Further characterization of the spent catalysts yielded a slight decrease in specific surface area for PDC1400 (from 640 m^2^ g^−1^ to 540 m^2^ g^−1^) and a slight increase for PDC1500 (from 182 m^2^ g^−1^ to 209 m^2^ g^−1^) (Figure 2 a). The microstructure of the PDC materials, probed by TPO (Figure 2 b), Raman spectroscopy (Figures S6–S9), XRD (Figures 2 c,d), and TEM (Figures 1 d, S14) did not appear to be influenced significantly by the chosen reaction conditions. Furthermore, the state of hybridization (sp^2^ fraction) as determined by electron energy loss spectroscopy (EELS) mapping (Figures S20 and S21) and X‐ray photoelectron spectroscopy (XPS, Figures S22 and S23) is very similar for the used catalysts PDC1400‐AR and PDC1500‐AR (see side note S2 and Table S2 in SI for detailed discussion on the characterization of the spent PDC catalysts).

Besides structural features, the in situ formed surface functionalization is believed to play a major role and ketonic/quinoidic carbonyl and phenolic surface groups are understood to be the relevant groups for ODH reactions on carbon catalysts.[^3^, ^7^, ^11^, ^19^] Temperature‐programmed desorption (TPD) of the polymer‐derived carbons reveals that PDC1400‐AO and PDC1400‐AR possess a significantly higher overall amount of oxygen surface groups compared to PDC1500‐AO and PDC1500‐AR (Figure S24). However, analysis of the TPD CO emission profile of PDC1400‐AR and PDC1500‐AR shows that PDC1500‐AR exhibits higher concentrations (229 μmol g^−1^ for PDC1500‐AR vs. 173 μmol g^−1^ for PDC1400‐AR) of a high‐temperature‐stable (emission maximum ca. 980 °C) CO emitting surface species that is associated with quinones (Figure S25).[^30^, ^31^] This finding is supported by the analysis of the XPS O1s region which, in case of PDC1500‐AR, shows a higher contribution of an oxygen species which exhibits a binding energy of around 530 eV compared to PDC1400‐AR (2.2 at % for PDC1400‐AR vs. 2.6 at % for PDC1500‐AR, Figure S26). The region of this binding energy is associated with the presence of surface ketones/quinones.^[31]^ However, it should be noted at this point that the observed differences in the surface oxide profiles between PDC1400‐AR and PDC1500‐AR are by far not as significant as the differences in catalytic performance, which indicates the presence of a high number of spectator species that do not contribute to the catalytic activity of the carbon material.

Considering the far higher specific surface area (540 vs. 209 m^2^ g^−1^) and higher number of oxygen surface groups of PDC1400‐AR, the only differences that speak for PDC1500‐AR as the “better” dehydrogenation catalyst are found to be the presence of a graphitic phase of higher stacking order or, to be more exact, of a stacking distance closer to ideal graphite, and small deviations in the surface oxide profile. In light of the evidence for the presence of a high number of spectator species, we want to propose the hypothesis that the carbon backbone of a suitable surface oxide plays a crucial role in the redox activity of the functional group, and differentiates an active site from a spectator. Hence, even the presence of a high number of the “right” oxygen surface functionality may not translate into catalytic activity if these groups are located on an unsuitable carbon backbone (see side note S3 for further discussion).

Strikingly, PDC1400 and PDC1500 exhibit fundamental differences even though the synthesis temperature only differs by 100 °C. As this behavior could be reproduced by several PDC batches, our current hypothesis relies on a phase transition of the Ni graphitization catalyst in the range between 1400 and 1500 °C. Macroscopically, Ni features a melting point at 1455 °C, which falls directly in between the utilized synthesis temperatures. Molten Ni might exhibit different graphitization characteristics compared to conventional Ni particles, leading to the observed differences in carbon microstructure as well as texture and finally to superior catalytic performance. This phenomenon is not limited to Ni as graphitization catalyst, but also applies to Co. Underscoring the presented phase‐transition hypothesis, Co also features a melting point between 1400 and 1500 °C, which translates into graphitic domains of stacking distances close to ideal graphite when Co is employed to graphitize PDC at 1500 °C (Figure S27a). Furthermore, PDC graphitized with Co at 1500 °C showed a similarly high catalytic performance as Ni‐graphitized PDC1500‐AO (Figure S27b).

**Figure 4:**
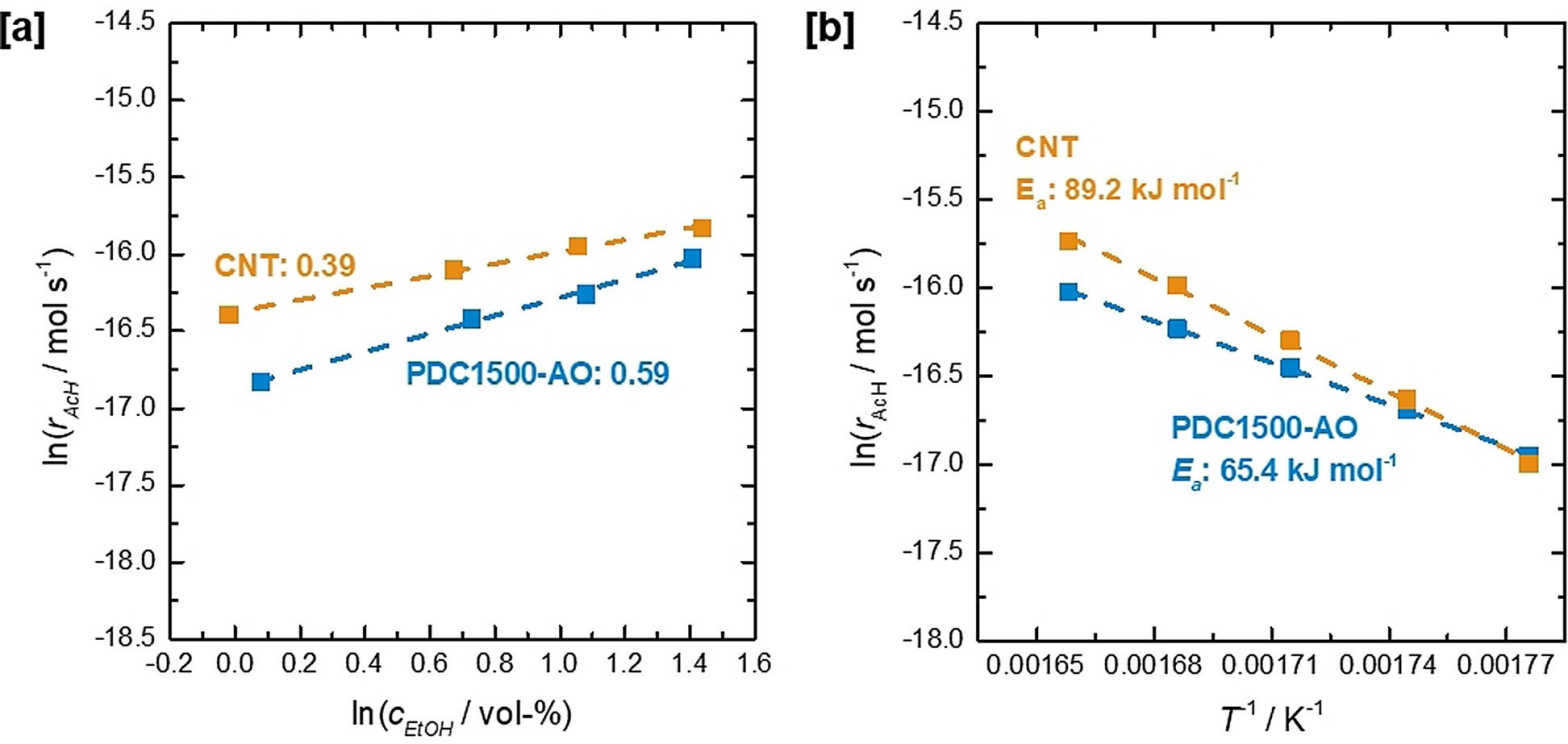
[a] Determination of the reaction order of ethanol for the formation of AcH for PDC1500‐AO and CNT catalysts. [b] Arrhenius plot for the determination of apparent activation energies for the formation of AcH for PDC1500‐AO and the CNT benchmark catalyst.

In order to obtain further catalytic insights for PDC1500‐AO and the CNT benchmark, a detailed (macro‐)kinetic study was carried out, varying oxygen concentration, ethanol concentration, and temperature during steady‐state experiments (Figure S28). It needs to be noted that influences of pore diffusion limitation could be ruled out by consulting the Prater–Weisz criterion (see Equations S1–3, Table S3). Kinetics of consumption of oxygen and ethanol were found to follow approximately a power‐law approach within the investigated concentration ranges. In light of similar surface oxide profiles, the reaction orders for the consumption of oxygen were found to be 0.26 for both PDC1500‐AO and for the CNT catalyst, hinting at similar mechanisms for O_2_ activation (Figures S26 and S29). Reaction orders for ethanol were determined to be 0.59 and 0.39, while the apparent activation energies for acetaldehyde formation were found to be 65.4 kJ mol^−1^ and 89.2 kJ mol^−1^ for PDC1500‐AO and the CNT catalyst, respectively (Figure 4). The non‐integer reaction orders hint at a complex network of elementary reactions being responsible for the observed kinetic behavior, which needs to be clarified in further studies.

## Conclusion

We propose a synthesis route towards a new generation of carbon‐based dehydrogenation catalysts based on amorphous/graphitic hybrid materials obtained by catalytic graphitization of a polymer precursor. The active catalyst is prepared by removing the amorphous matrix of the hybrid materials by mild oxidation, thereby creating access to the previously grown graphitic domains. These graphitic domains are disordered on a mesoscopic scale and rich in defects, providing a suitable carbon backbone for highly redox‐active oxygen surface groups. A reaction temperature above 1500 °C appears to be crucial to the synthesis of an active, selective, and stable PDC catalyst. While showing the same excellent selectivity at similar conversion in the ODH of ethanol as carbon nanotubes, polymer‐derived carbons synthesized at 1500 °C outperform the CNT benchmark in terms of space–time yield by nearly one order of magnitude. In addition to superior catalytic performance, the next‐generation PDC materials are accessible via a scalable synthetic pathway and exhibit spherical particles with diameters around 100 μm. There are several conceivable strategies that could broaden the future impact of these new carbon catalysts. Beyond Ni, several transition metals (eg. Co, Fe) can serve as graphitization catalyst, and the influence of the nature and loading of the graphitization catalyst on microstructure and catalytic performance could be studied. Furthermore, doping with heteroatoms such as N, S, and P could be employed to tailor the electronic properties (redox activity) and surface chemistry of PDC materials. On the one hand, the polymer‐based synthesis strategy enables a direct, controlled heteroatom doping by copolymerisation while, on the other hand, simple post‐synthesis doping methods promise to be feasible as well. Finally, the scope of applications for the new PDC catalysts could be extended to include the oxidative dehydrogenation of various other relevant substrates such as alkanes and alcohols beyond ethanol, as well as to electro‐ and photocatalysis.

## Conflict of interest

The authors declare no conflict of interest.

## Supporting information

As a service to our authors and readers, this journal provides supporting information supplied by the authors. Such materials are peer reviewed and may be re‐organized for online delivery, but are not copy‐edited or typeset. Technical support issues arising from supporting information (other than missing files) should be addressed to the authors.

Supplementary

## References

[1] G. J. Hutchings , J. Mater. Chem. 2009, 19, 1222.

[2a] RSC green chemistry, Vol. 28 (Eds.: F. Cardona , C. Parmeggiani ), The Royal Society of Chemistry, Cambridge, 2015;

[2b] F. Cavani , N. Ballarini , A. Cericola , Catal. Today 2007, 127, 113;

[2c] A. W. Franz , H. Kronemayer , D. Pfeiffer , R. D. Pilz , G. Reuss , W. Disteldorf , A. O. Gamer , A. Hilt in Ullmann's Encyclopedia of Industrial Chemistry, Wiley-VCH, Weinheim, 2000, pp. 1–34;

[2d] D. H. James , W. M. Castor in Ullmann's Encyclopedia of Industrial Chemistry, Wiley-VCH, Weinheim, 2000;

[2e] T. Ohara , T. Sato , N. Shimizu , G. Prescher , H. Schwind , O. Weiberg , K. Marten , H. Greim in Ullmann's Encyclopedia of Industrial Chemistry, Wiley-VCH, Weinheim, 2000.

[3] G. Emig , H. Hofmann , J. Catal. 1983, 84, 15.

[4] T. G. Alkhazov , A. E. Lisovskii , T. K. Gulakhmedova , React. Kinet. Catal. Lett. 1979, 12, 189.

[5] B. Frank , M. Morassutto , R. Schomäcker , R. Schlögl , D. S. Su , ChemCatChem 2010, 2, 644.

[6] B. Frank , J. Zhang , R. Blume , R. Schlögl , D. S. Su , Angew. Chem. Int. Ed. 2009, 48, 6913; 10.1002/anie.200901826 19670279

[7] J. Zhang , X. Liu , R. Blume , A. Zhang , R. Schlögl , D. S. Su , Science 2008, 322, 73. 18832641 10.1126/science.1161916

[8] J. Zhang , D. Su , A. Zhang , D. Wang , R. Schlogl , C. Hebert , Angew. Chem. Int. Ed. 2007, 46, 7319; 10.1002/anie.200702466 17722129

[9a] J. Wang , R. Huang , Z. Feng , H. Liu , D. Su , ChemSusChem 2016, 9, 1820; 27282126 10.1002/cssc.201600234

[9b] R. D. Weinstein , A. R. Ferens , R. J. Orange , P. Lemaire , Carbon 2011, 49, 701;

[9c] J. Wang , R. Huang , Y. Zhang , J. Diao , J. Zhang , H. Liu , D. Su , Carbon 2017, 111, 519;

[9d] G. C. Grunewald , R. S. Drago , J. Am. Chem. Soc. 1991, 113, 1636.

[10] M. Pereira , J. Órfão , J. L. Figueiredo , Appl. Catal. A 1999, 184, 153.

[11] G. Mestl , N. I. Maksimova , N. Keller , V. V. Roddatis , R. Schlögl , Angew. Chem. Int. Ed. 2001, 40, 2066; 10.1002/1521-3773(20010601)40:11<2066::AID-ANIE2066>3.0.CO;2-I 29712216

[12] M. Pereira , J. Órfão , J. Figueiredo , Appl. Catal. A 2001, 218, 307.

[13] T.-J. Zhao , W.-Z. Sun , X.-Y. Gu , M. Rønning , D. Chen , Y.-C. Dai , W.-K. Yuan , A. Holmen , Appl. Catal. A 2007, 323, 135.

[14] N. Keller , N. I. Maksimova , V. V. Roddatis , M. Schur , G. Mestl , Y. V. Butenko , V. L. Kuznetsov , R. Schlögl , Angew. Chem. Int. Ed. 2002, 41, 1885; 10.1002/1521-3773(20020603)41:11<1885::aid-anie1885>3.0.co;2-5 19750622

[15] V. Schwartz , W. Fu , Y.-T. Tsai , H. M. Meyer , A. J. Rondinone , J. Chen , Z. Wu , S. H. Overbury , C. Liang , ChemSusChem 2013, 6, 840. 23471876 10.1002/cssc.201200756

[16] J. J. Delgado , X. Chen , J. P. Tessonnier , M. E. Schuster , E. Del Rio , R. Schlögl , D. S. Su , Catal. Today 2010, 150, 49.

[17] D. Su , N. I. Maksimova , G. Mestl , V. L. Kuznetsov , V. Keller , R. Schlögl , N. Keller , Carbon 2007, 45, 2145.

[18a] M. F. R. Pereira , J. L. Figueiredo , J. J. Órfão , P. Serp , P. Kalck , Y. Kihn , Carbon 2004, 42, 2807;

[18b] X. Guo , W. Qi , W. Liu , P. Yan , F. Li , C. Liang , D. Su , ACS Catal. 2017, 7, 1424.

[19] W. Qi , W. Liu , X. Guo , R. Schlögl , D. Su , Angew. Chem. Int. Ed. 2015, 54, 13682; 10.1002/anie.201505818 26388451

[20a] N. Xiao , Y. Zhou , Z. Ling , Z. Zhao , J. Qiu , Carbon 2013, 60, 514;

[20b] W. Qi , D. Su , ACS Catal. 2014, 4, 3212.

[21] J. Gläsel , J. Diao , Z. Feng , M. Hilgart , T. Wolker , D. S. Su , B. J. M. Etzold , Chem. Mater. 2015, 27, 5719.

[22] J. Hoekstra , A. M. Beale , F. Soulimani , M. Versluijs-Helder , J. W. Geus , L. W. Jenneskens , J. Phys. Chem. C 2015, 119, 10653.

[23] A. Ōya , H. Marsh , J. Mater. Sci. 1982, 17, 309.

[24] A.-H. Lu , W.-C. Li , E.-L. Salabas , B. Spliethoff , F. Schüth , Chem. Mater. 2006, 18, 2086.

[25] S.-H. Chai , J. Y. Howe , X. Wang , M. Kidder , V. Schwartz , M. L. Golden , S. H. Overbury , S. Dai , D.-e. Jiang , Carbon 2012, 50, 1574.

[26a] M. Eckert , G. Fleischmann , R. Jira , H. M. Bolt , K. Golka in Ullmann's Encyclopedia of Industrial Chemistry, Wiley-VCH, Weinheim, 2000;

[26b] N. Kosaric , Z. Duvnjak , A. Farkas , H. Sahm , S. Bringer-Meyer , O. Goebel , D. Mayer in Ullmann's Encyclopedia of Industrial Chemistry, Wiley-VCH, Weinheim, 2000, pp. 1–72;

[26c] A. J. Ragauskas , C. K. Williams , B. H. Davison , G. Britovsek , J. Cairney , C. A. Eckert , W. J. Frederick, Jr. , J. P. Hallett , D. J. Leak , C. L. Liotta , et al., Science 2006, 311, 484. 16439654 10.1126/science.1114736

[27] M. Thommes , K. Kaneko , A. V. Neimark , J. P. Olivier , F. Rodriguez-Reinoso , J. Rouquerol , K. S. Sing , Pure Appl. Chem. 2015, 87, 1051.

[28] H. Fujimoto , Carbon 2003, 41, 1585.

[29] A. V. Alaferdov , R. Savu , M. A. Canesqui , Y. V. Kopelevich , R. R. da Silva , N. N. Rozhkova , D. A. Pavlov , Y. Usov , G. M. de Trindade , S. A. Moshkalev , Carbon 2018, 129, 826.

[30a] J. Figueiredo , M. Pereira , M. Freitas , J. Órfão , Carbon 1999, 37, 1379;

[30b] J. L. Figueiredo , M. F. R. Pereira , M. M. A. Freitas , J. J. M. Órfão , Ind. Eng. Chem. Res. 2007, 46, 4110.

[31] K. Friedel Ortega , R. Arrigo , B. Frank , R. Schlögl , A. Trunschke , Chem. Mater. 2016, 28, 6826.

