# Supplementary material for: Nanoscale Hybrid Amorphous/Graphitic Carbon as Key Towards Next‐Generation Carbon‐Based Oxidative Dehydrogenation Catalysts
Source: Angew Chem Int Ed Engl. 2021 Jan 26;60(11):5898–906. doi: 10.1002/anie.202014862 (PMC7986892; doi:10.1002/anie.202014862)
Supplement: Supplementary file 1 — Supplementary [file ANIE-60-5898-s001.pdf]

Angewandte Chemie
Eine Zeitschrift der Gesellschaft Deutscher Chemiker

Supporting Information

Nanoscale Hybrid Amorphous/Graphitic Carbon as Key Towards Next-Generation Carbon-Based Oxidative Dehydrogenation Catalysts

*Felix Herold[+], Stefan Prosch[+], Niklas Oefner, Kai Brunnengräber, Oliver Leubner, Yannick Hermans, Kathrin Hofmann, Alfons Drochner, Jan P. Hofmann, Wei Qi, and Bastian J. M. Etzold**

anie_202014862_sm_miscellaneous_information.pdf

Supplementary Information

METHODS

Synthesis of polymer derived carbon (PDC)

Precursor polymer

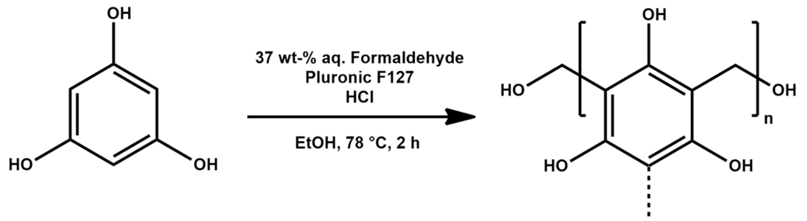

Scheme S1: Polymerization of 1,3,5-trihydroxy benzene with formaldehyde in presence of the soft template Pluronic F127.

To obtain pristine polymer particles, a synthesis procedure following CHAI et al. was applied.[1] In short, in a 3 L round bottom flask, 26.2 g Phloroglucinol (1,3,5-trihydroxy benzene, Acros Organics), 52.4 g Pluronic® F127 (Sigma Aldrich) and 10 g 37 wt-% HCl (Fisher Scientific) are heated under stirring with a KPG stirrer (500 rpm, half-moon impeller, 70 mm blade) in 1320 mL EtOH (Fisher Scientific) to reflux. Upon addition of 26 g 37 wt-% aqueous Formaldehyde (Sigma Aldrich) solution, the reaction mixture turns turbid within minutes. After refluxing for 2 h, a yellow precipitate is collected by filtration. After washing thoroughly with EtOH and vacuum drying at 30 mbar and 60 °C, 47.3 g of bright yellow polymer aggregates are obtained.

Carboxyl-Polymer

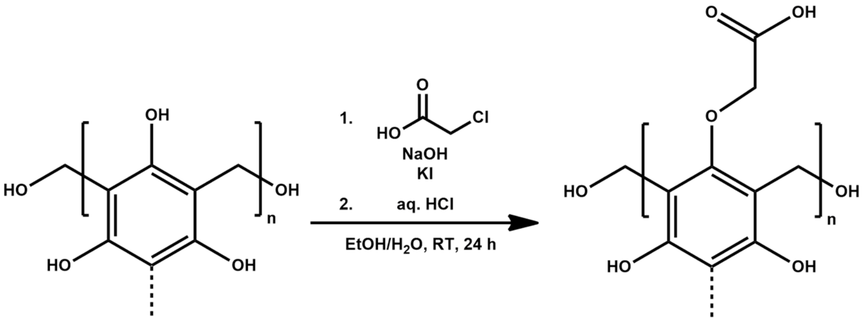

Scheme S2: Conversion of phenolic hydroxy groups of the precursor polymer with chloroacetic acid.

45 g of the precursor polymer and 4.2 g potassium iodide (Fisher Scientific) are suspended in 1300 ml EtOH (Fisher Scientific). Under stirring with a KPG stirrer, 75 g NaOH (Acros Organics) in 400 mL H_2O are slowly added to the reaction mixture. Over the course of 1 h, a mixture of 75 g of chloroacetic acid (Acros Organics) and 31 g NaOH in 250 mL H_2O is added dropwise under stirring to the reaction suspension. After stirring for additional 23 h at room temperature, the polymer particles are collected by filtration and washed thoroughly with EtOH and H_2O. By drying at 30 mbar and 60 °C, 44 g of red-brown polymer aggregates are obtained.

Loading of Ni via ion exchange

Initially, in order to load the carboxyl-polymer with H^+-ions, 40 g of the polymer particles are suspended in 500 mL 10 wt-% HCl (Fisher Scientific) for 30 min on an orbital shaker. Subsequently, the red polymer aggregates are filtrated, washed with deionized H_2O, and suspended again in 500 mL of a 1 M solution of nickel(II) chloride hexahydrate (Alfa Aesar) in deionized H_2O for 24 h at room temperature on an orbital shaker. Afterwards, the Ni-loaded polymer particles are filtrated, washed with deionized H_2O and dried at 30 mbar and 60 °C.

Pyrolysis

20 g Ni-loaded polymer particles are subjected to pyrolysis in a horizontal tubular furnace (Carbolite Gero GmbH) under a flow of 20 L/h (STP) He (all gases were purchased from

Westfalen AG). The samples are heated at a rate of 120 K/h, the target temperature (here 1400 to 1500 °C) is hold for 2 h, subsequently the samples are cooled to room temperature with a cooling rate of 120 K/h.

In order to remove the graphitization catalyst, 8 g of Ni-loaded carbon are suspended in 100 mL of 18.5 wt-% HCl (Fisher Scientific) and stirred at 90 °C for 24 h. Subsequently, the polymer-derived carbon is collected by filtration and washed thoroughly with deionized H_2O. This acid washing procedure is carried out three times. Afterwards, the carbon particles are dried at 30 mbar and 60 °C.

Oxidation

500 mg of polymer-derived carbon are loaded in a U-shaped tubular quartz reactor with an internal diameter of 4 mm and fixed on both ends with quartz wool. The sample is heated under a volume flow of 20 mL/min (STP) He to 380 °C, and the He atmosphere is replaced with synthetic air at the same volume flow. After 72 h, the atmosphere is switched back to He, and the sample is allowed to cool. The CNT benchmark catalyst was preoxidized using the same procedure.

Catalytic testing

Catalytic reactions were performed in a continuous flow apparatus with a quartz U-tube with an internal diameter of 4 mm in which the catalyst (~ 90 mg) was placed between two glass wool plugs. Ethanol was fed to the reactor via a two-stage saturator system, gas dosing was realized by mass flow controllers. Typical experiments were conducted at 330 °C, while 4.3 vol-% EtOH (HPLC-grade, Fisher Scientific), 10 vol-% O_2 at 20 mL/min (STP) total volume flow with He as inert gas were used. Off-gas analytic was performed by online mass spectrometry (GAM 400, InProcess Instruments).

Analytical Procedures

Potentiometric titrations were conducted using an automatic titrator Mettler Toledo T50. About 80 mg of the sample were suspended in 60 mL of a 0.1 M aqueous solution of KNO_3 (Fisher Scientific) and adjusted to an initial value of pH 3 with 0.1 M HCl (Merck). The actual titration was carried out with 0.1 M KOH (Merck), dosing 50 µL every 1200 s while bubbling N_2 through the suspension in order to avoid carbonate formation. Diffuse reflectance infrared Fourier transform spectroscopy (DRIFTS) was performed in a Bruker VERTEX 70 spectrometer with a resolution of 2 cm^{-1} using a Praying-Mantis diffuse reflection accessory (Harrick Scientific) and optical grade KBr (Acros Organics) as reference. Raman spectroscopy was carried out in a Raman microscope of the type Bruker Senterra I using a Nd:YAG laser with an excitation wavelength of 532 nm at 0.5 mW with a resolution of 1 cm^{-1}, an integration period of 2 s and 30 co-additions. The fitting of the Raman spectra and extraction of I_D/I_G ratios was performed according to MALLET-LADEIRA et al.[2] Of each sample, at least 10 Raman spectra were recorded and evaluated. Transmission electron microscopy was carried out with a JEOL JEM-2100F device at an acceleration voltage of 200 kV. STEM-EELS maps were acquired on an ARM-200F operated at 200 keV and equipped with a Gatan Image Filter (GIF). EELS-Maps were measured with an energy offset of 210.0 eV and dispersion of 0.2 eV. Every pixel was measured with an exposure time of 1.0s. sp^2/sp^3-ratios were calculated from the EELS maps applying the method described by BERNIER *et al.*.[3] TEM-analysis required grinding of the samples. Field emission scanning electron microscopy images were taken using a Philips XL30 FEG electron microscope. Samples were sputtered with gold for 300 s at a potential of 30 mV. Pictures of polymers and carbon were taken at an acceleration voltage of 10 to 15 kV. Thermogravimetric measurements were conducted in a STA 449 Jupiter thermogravimetry device (NETZSCH GmbH) using a 60 mL/min (STP) N_2 purge a heating rate of 5 K/min and a sample mass of ~20 mg. Temperature programmed oxidation (TPO) measurements were carried out in a STA 449 Jupiter thermogravimetry device using 50 mL/min (STP) synthetic air, a heating rate of 5 K/min and a sample mass of ~20 mg. Temperature programmed

desorption (TPD) was conducted in a STA 409PC Luxx thermogravimetry device (NETZSCH GmbH) coupled to an online mass spectrometer (Omnistar, Pfeiffer Vacuum GmbH). Under a stream of 30 mL/min (STP) He, 50 mg of a carbon sample were initially heated with a rate of 5 K/min to 100 °C, where the sample was dried for 2 h. Subsequently, the sample was heated from 100 °C to 1000 °C with a rate of 5 K/min. Deconvolution of the TPD profiles was carried out building on work of PEREIRA, FIGUEIREDO and co-workers by utilizing Gaussian functions and a least squares fit procedure.[4] In case of the CO_2 emission profile, the temperature maximum of carboxylic acids was positioned at 310 ± 25 °C, that of anhydrides at 450 ± 25°C and that of lactones at 650 ± 25 °C. In a similar manner, deconvolution of the CO emission profiles was carried out assuming the position of the desorption maxima of primary alcohols at 410 ± 25 °C, of phenols at 510 ± 25 °C, of carbonyl groups and ethers at 675 ± 25 °C, and two further carbonyl species at 825 ± 25 °C, and 975 ± 25 °C. The half width at half maximum (HWHM) was fixed for all contributions to a range of 75 ± 25 °C. N_2 - physisorption at -196 °C was conducted using a Quantachrome Quadrasorb device. Sample preparation included outgassing at 350 °C for 20 h at about 14 mTorr prior to physisorption experiments. Specific surface areas where determined using the BET method. X-ray photoelectron spectroscopy (XPS) measurements were carried out using a SPECS Phoibos 150 analyzer and a SPECS Focus 500 X-ray source using the monochromatized AlKα line at 1486.7 eV. The Fermi edge of a sputter cleaned silver sample was used to calibrate the binding energies of all photoelectron spectra. Pass energies of 25 eV and 10 eV were used for the survey and core level spectra, respectively. Spectra were evaluated using CasaXPS software. Powder X-ray diffraction measurements were carried out in a StadiP (Stoe & Cie GmbH) diffractometer in Debye-Scherrer geometry using $Cu_{K\alpha 1}$-radiation (λ = 1.54060 Å) in equipped with a Ge[111]-monochromator and a MYTHEN1K (Dectris Ltd.) detector. All samples were analysed in capillaries of an inner diameter of 0.5 mm. Inductive coupled plasma optical emission spectrometry (ICP-OES) was carried out using a PerkinElmer OPTIMA 2000DV spectrometer.

Sample preparation included the oxidation of the carbon sample in a muffle furnace and subsequent solubilisation of the residue in aqua regia.

POLYMER ANALYTICS

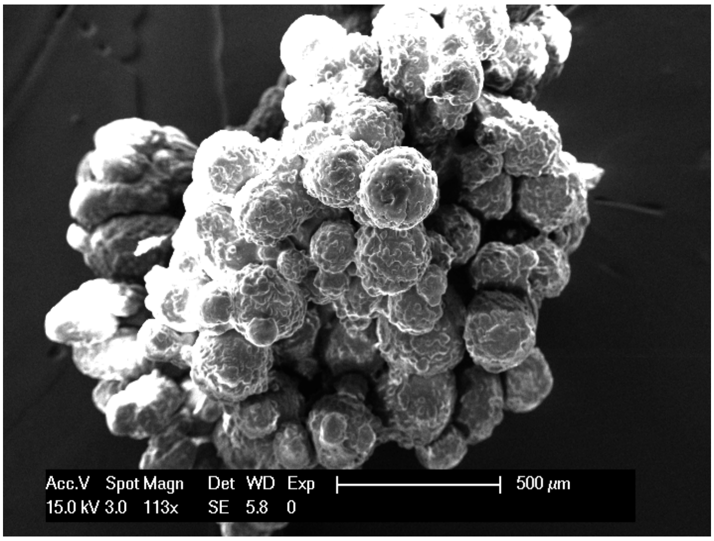

Figure S1: SEM-picture of an aggregate of particles of the precursor polymer.

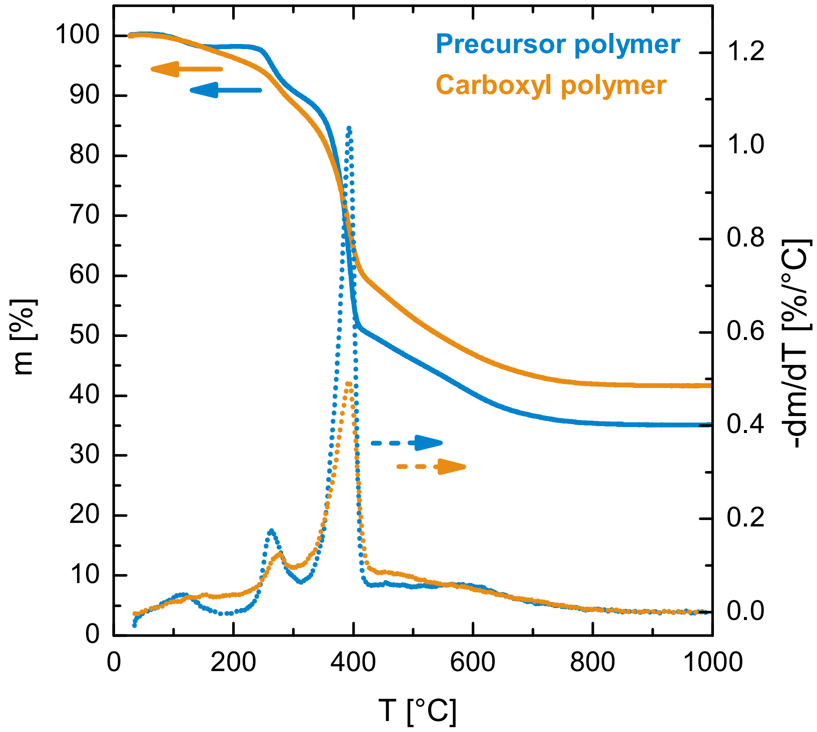

Figure S2: Thermogravimetric analysis of precursor polymer and carboxyl polymer (50 mg sample, 5 K min^{-1}, 60 ml min^{-1} (STP) N_2). The mass-loss at 400 °C corresponds to the thermal degradation of the PEO-PPO-PEO triblock copolymer soft-template Pluronic F127.[4]

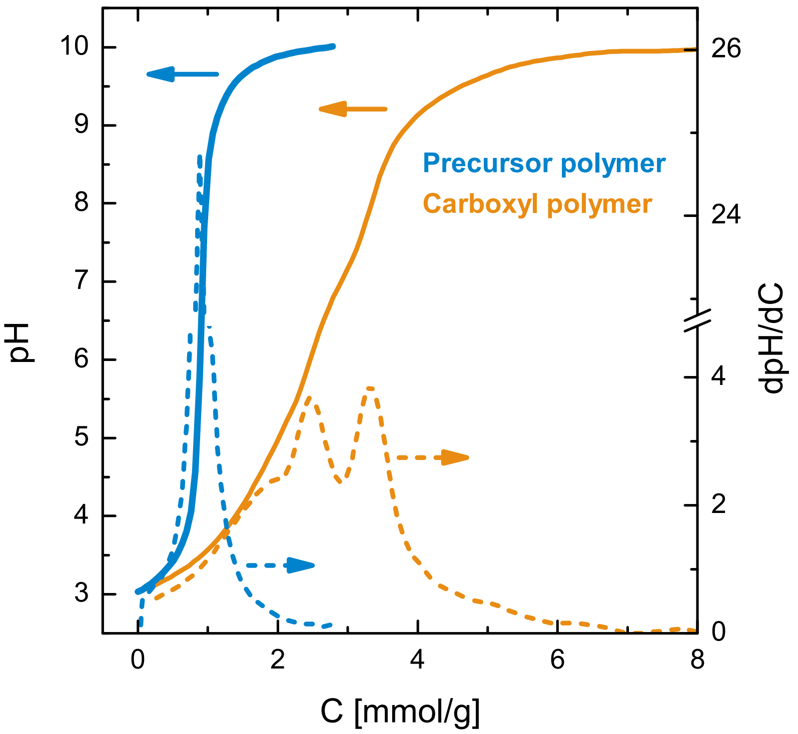

Figure S3: Potentiometric titration of precursor polymer and carboxyl polymer. The titration of the precursor polymer reveals a blank capacity of 0.89 mmol g^{-1} originating from dilution effects of the aqueous electrolyte. Furthermore, the presence of acidic phenolic hydroxyl groups is indicated by the distinct plateau in the interval of pH 9.5 to pH 10. The occurrence of two maxima in the first derivation of the titration curve of the carboxyl polymer may be assigned to the deprotonation of neighbouring carboxyl groups. The deprotonation of the first carboxyl group occurs at low pH values, the deprotonation of the second carboxylic acid appears to be hampered by the negative charge in close proximity. In consideration of the blank capacity, a capacity of 2.44 mmol g^{-1} can be determined for the carboxyl polymer from the second maximum of the derivation of the titration curve.

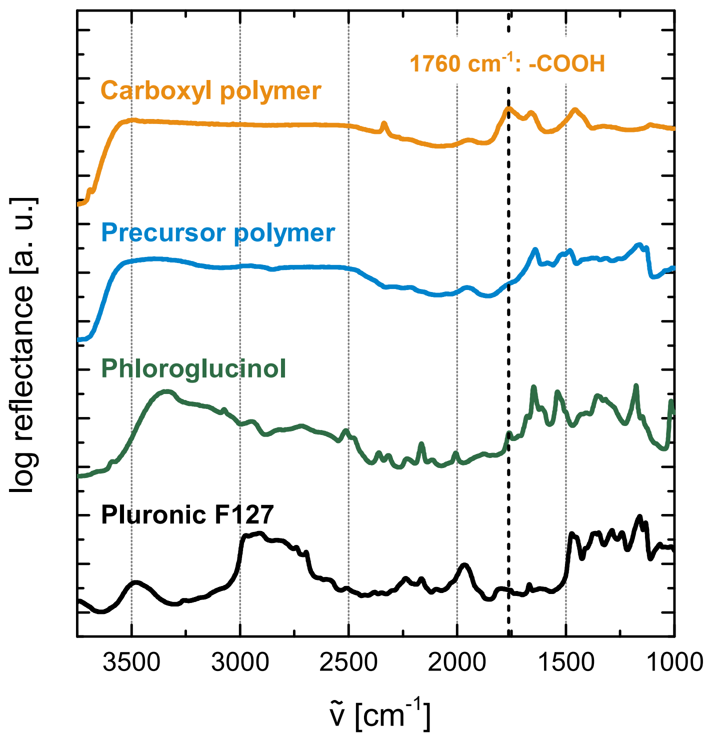

Figure S4: DRIFT – spectra of precursor polymer and carboxyl polymer as well as the main constituents phloroglucinol and Pluronic® F127 using KBr as reference physico-chemical.

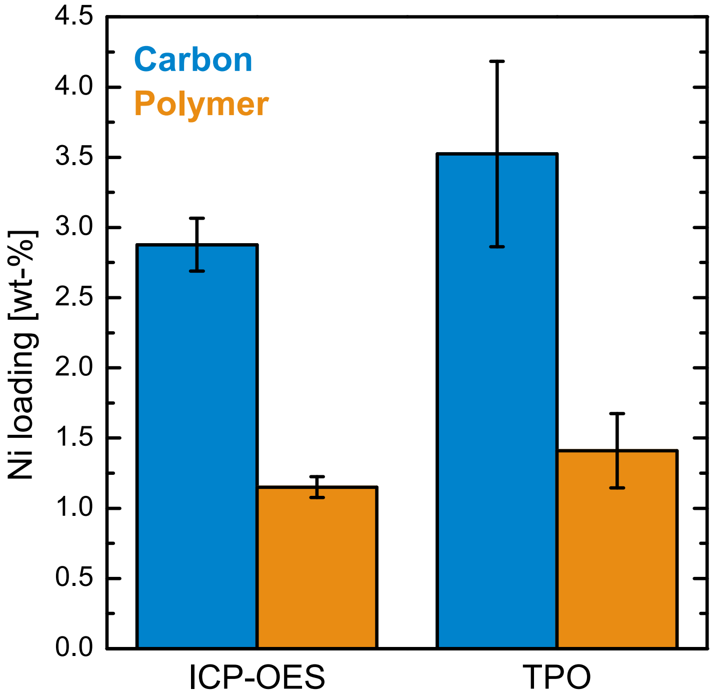

Figure S5: Loading of the Ni graphitization catalyst based on the mass of the carbon after pyrolysis and based on the mass of the polymer prior pyrolysis. The actual ICP-OES and TPO measurements were conducted using carbon samples after pyrolysis and prior acid leaching. The polymer loading was calculated using the pyrolysis yield (~ 40 wt-%). In case of TPO, NiO was assumed as sole component of the residue after oxidation.

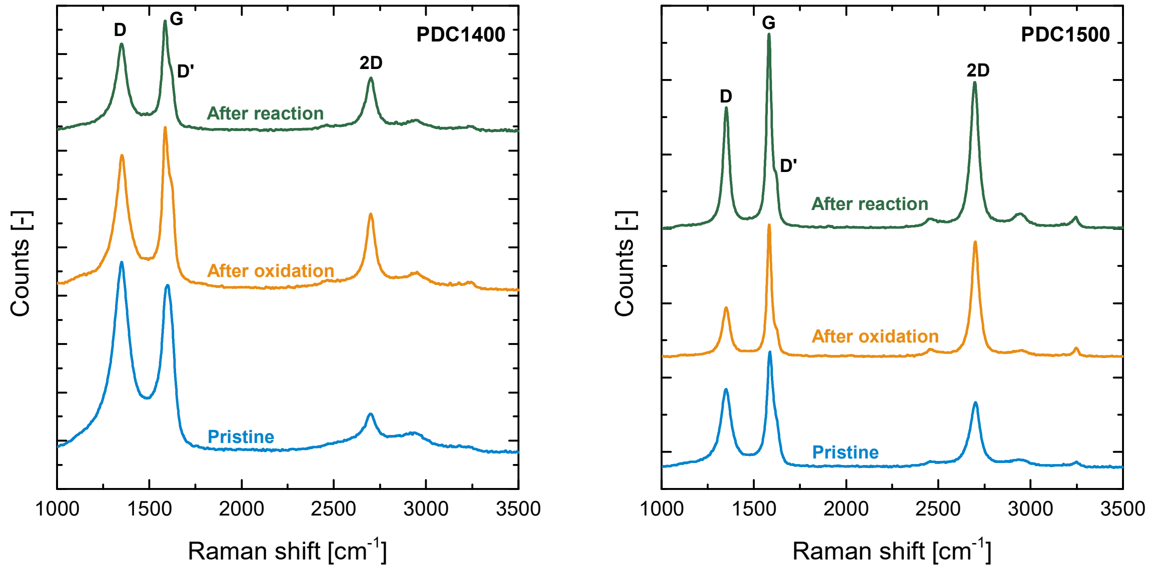

Figure S6: Representative Raman spectra of pristine PDC1400 and PDC1500 as well as after the synthetic air oxidation and after the reaction.

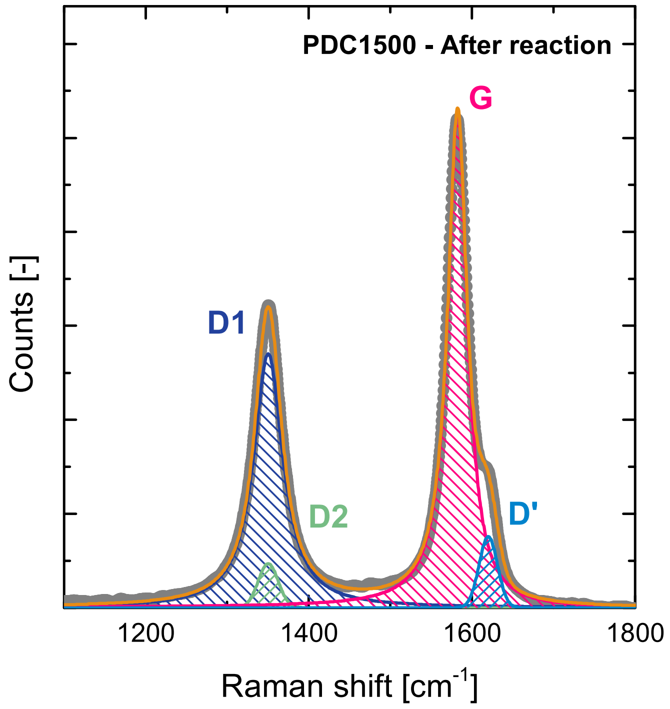

Figure S7: Representative fitting of a first order Raman spectrum of PDC1500 after reaction according to a procedure proposed by MALLET-LADEIRA et al.[2] I_D/I_G-ratios are obtained by addition of the intensity of D1 and D2 contributions.

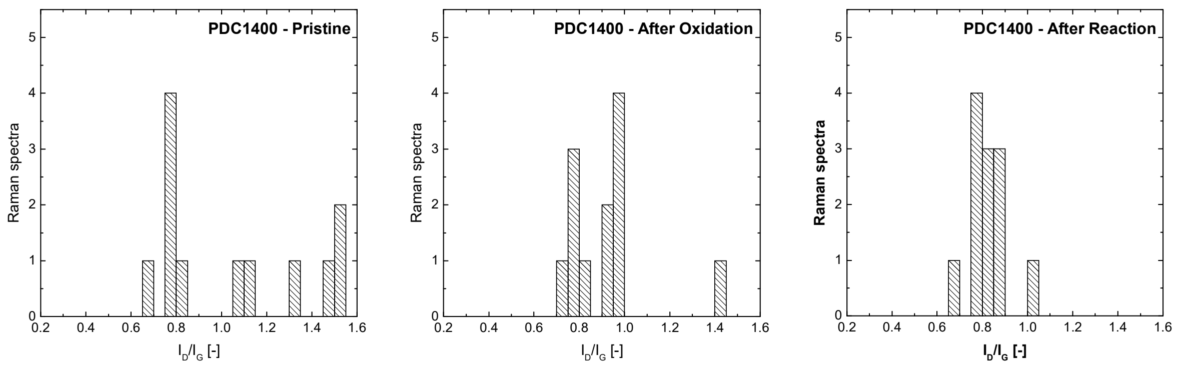

Figure S8: Number of Raman spectra of a given PDC1400 sample within a 0.05 interval of I_D/I_G ratio.

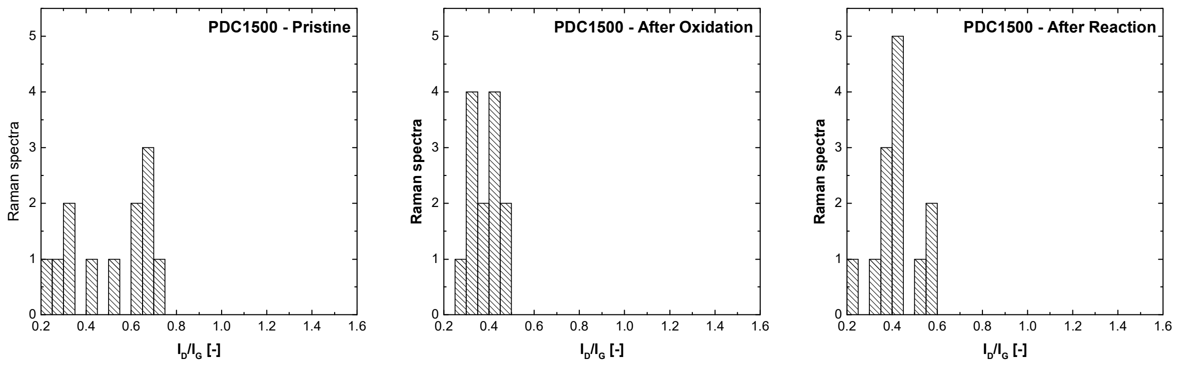

Figure S9: Number of Raman spectra of a given PDC1500 sample within a 0.05 interval of I_D/I_G ratio.

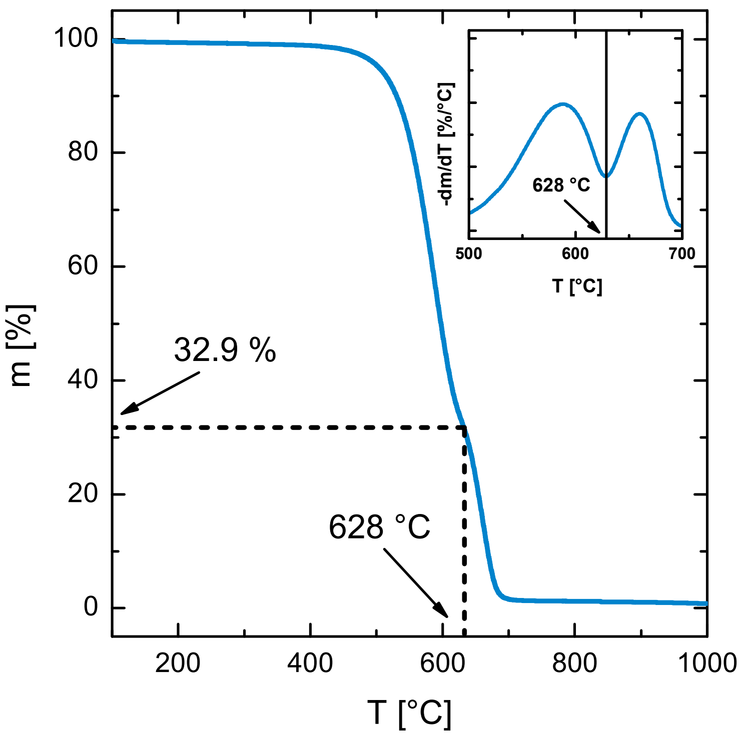

Figure S10: Identification of the residual mass at the infliction point of the mass loss curve of PDC1400-AO during temperature programmed oxidation. The minimum of the first derivation provides the temperature of the infliction point, the mass loss at this temperature can be extracted from the mass loss curve.

Table S1: Determination of the Ni content of PDC samples by ICP-OES.

**Sample**	**Ni-loading**
PDC1500-P (prior acid leaching)	2.9 ± 0.2 wt-%
PDC1500-P (after acid leaching)	0.041 ± 0.001 wt-%
PDC1500-AO-HCl (washed, oxidized and washed again with HCl)	below detection threshold

PDC1500-AO and PDC1500-AO-HCl (PDC1500-P which was washed, oxidized and washed with HCl again) showed matching steady state EtOH conversion (33 % for PDC1500-AO vs. 30 % for PDC1500-AO-HCl) and AcH selectivity (80 % for PDC1500-AO vs. 80 % for PDC1500-AO-HCl) after 20 h TOS.

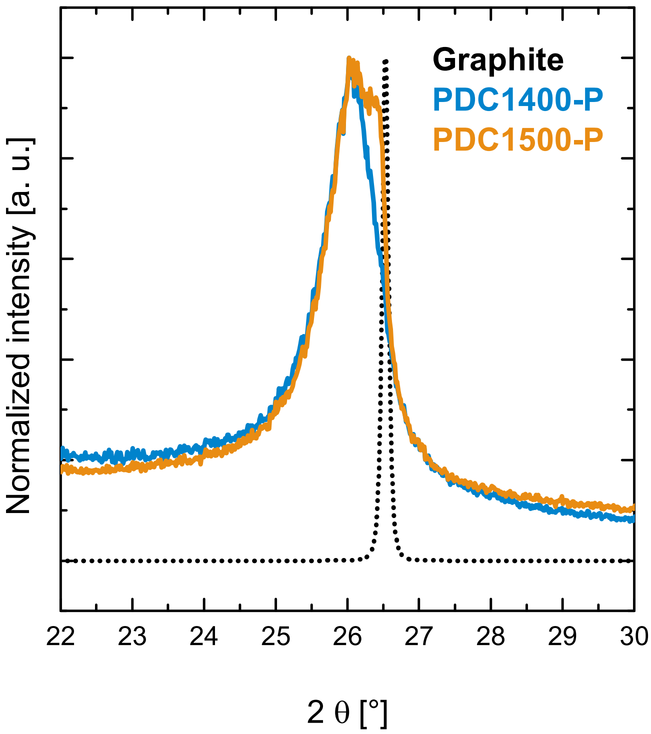

Figure S11: Detail of the (002) reflections of PDC1400-P, PDC1500-P compared to crystalline graphite-2H.

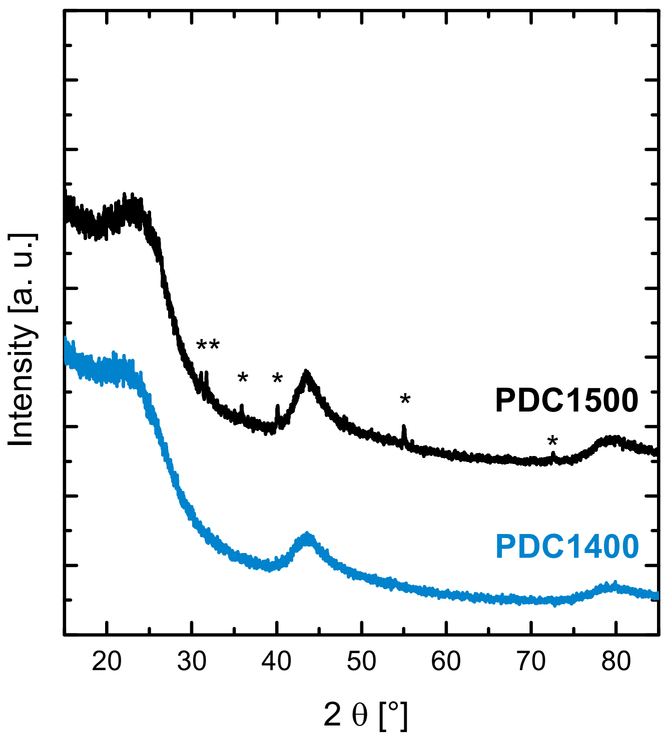

Figure S12: X-ray powder diffraction patterns of PDC1400 and PDC1500 pyrolyzed without Ni graphitization catalyst. Diffraction data of PDC1500 shows a contamination which can be assigned to Al_3C_4 (reflexes marked with asterisk), originating possibly from the Al_2O_3 crucible used for synthesis.

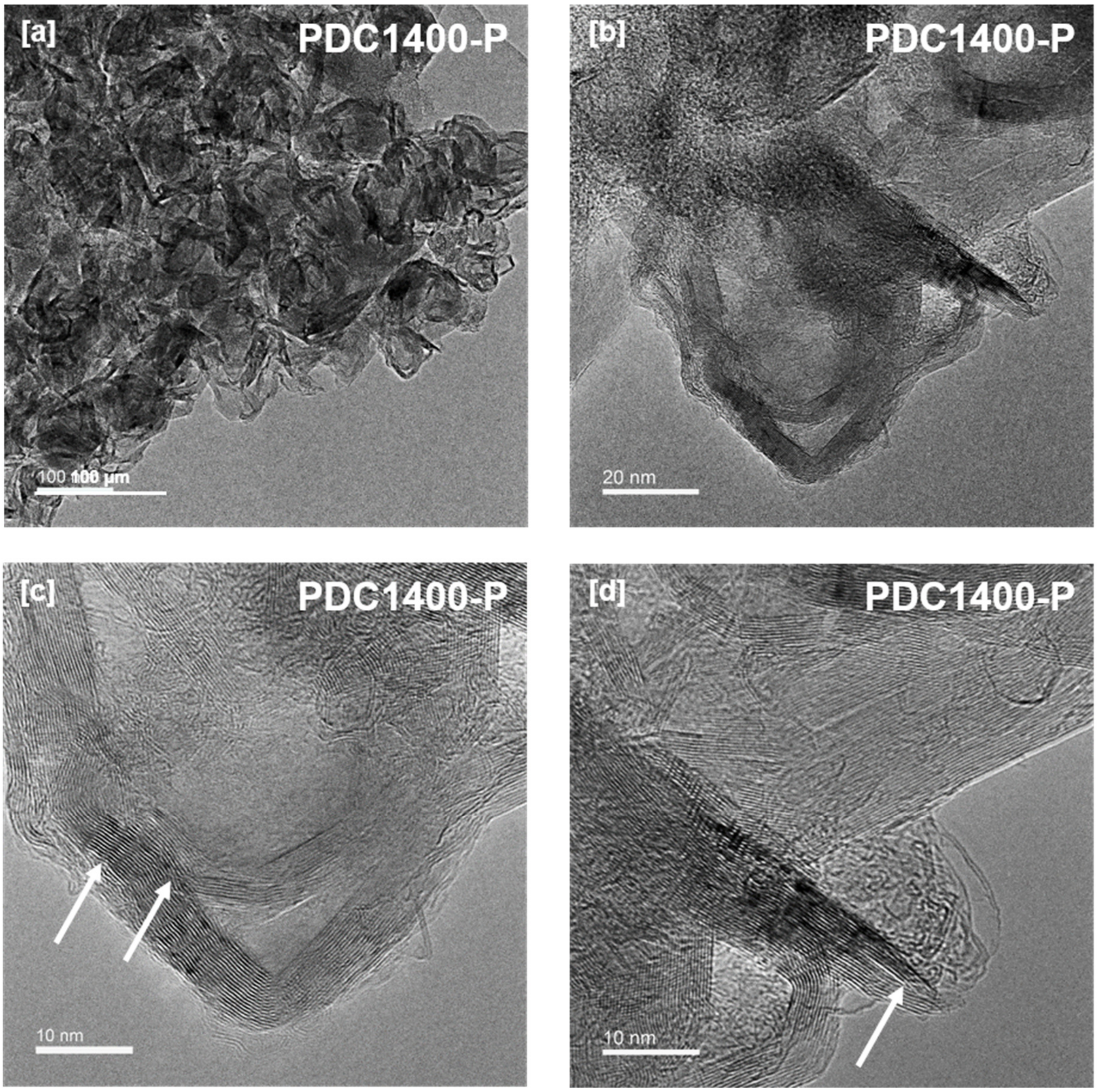

Figure S13: TEM micrographs of PDC1400-P in different magnifications [a,b,c,d]. The graphitic microstructure contains various defects such as buckling, merging and splitting graphene layers (see arrows) [c,d].

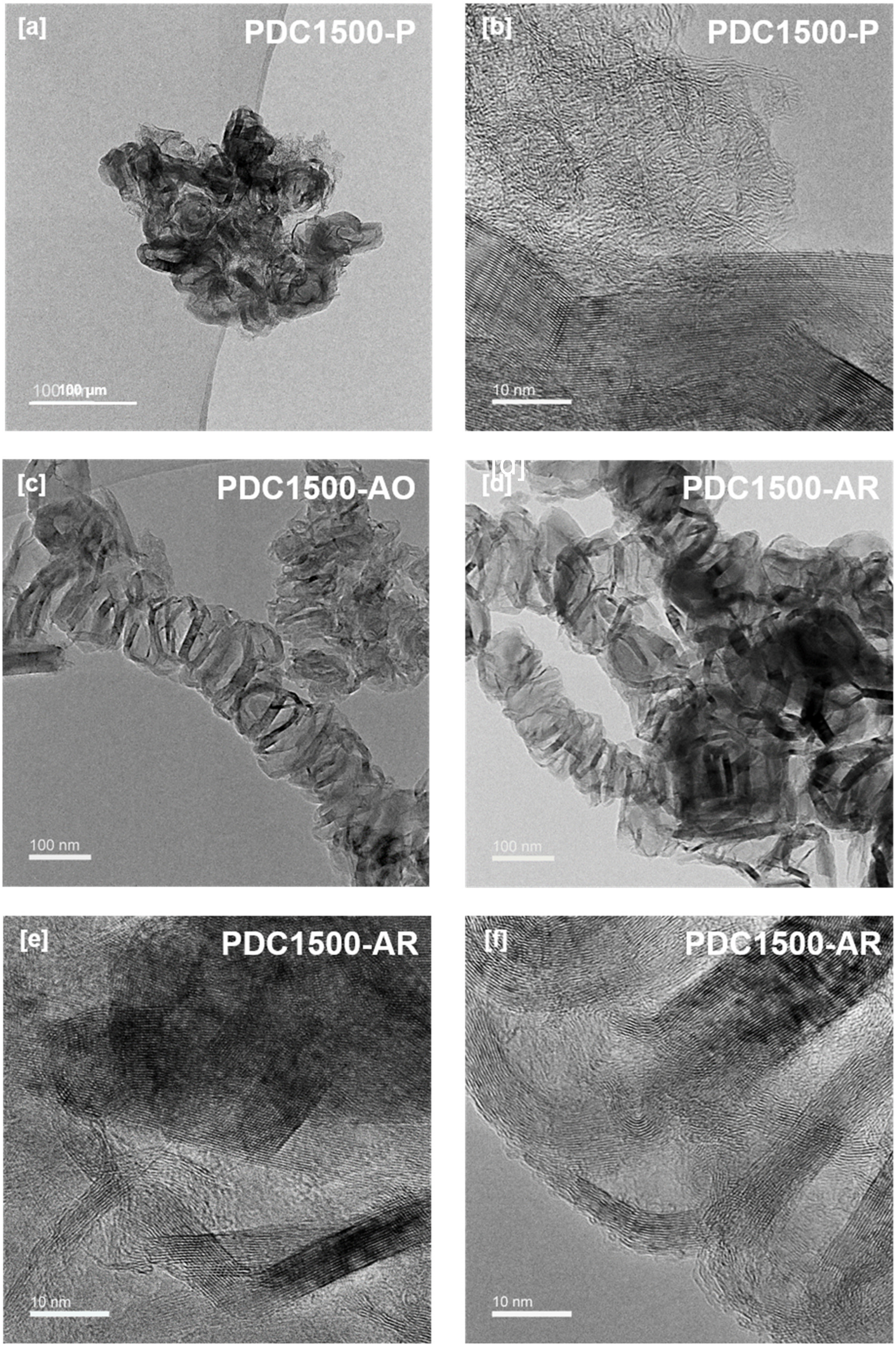

Figure S14: TEM micrographs of PDC1500-P [a,b], PDC1500-AO [c], PDC1500-AR [d,e,f].

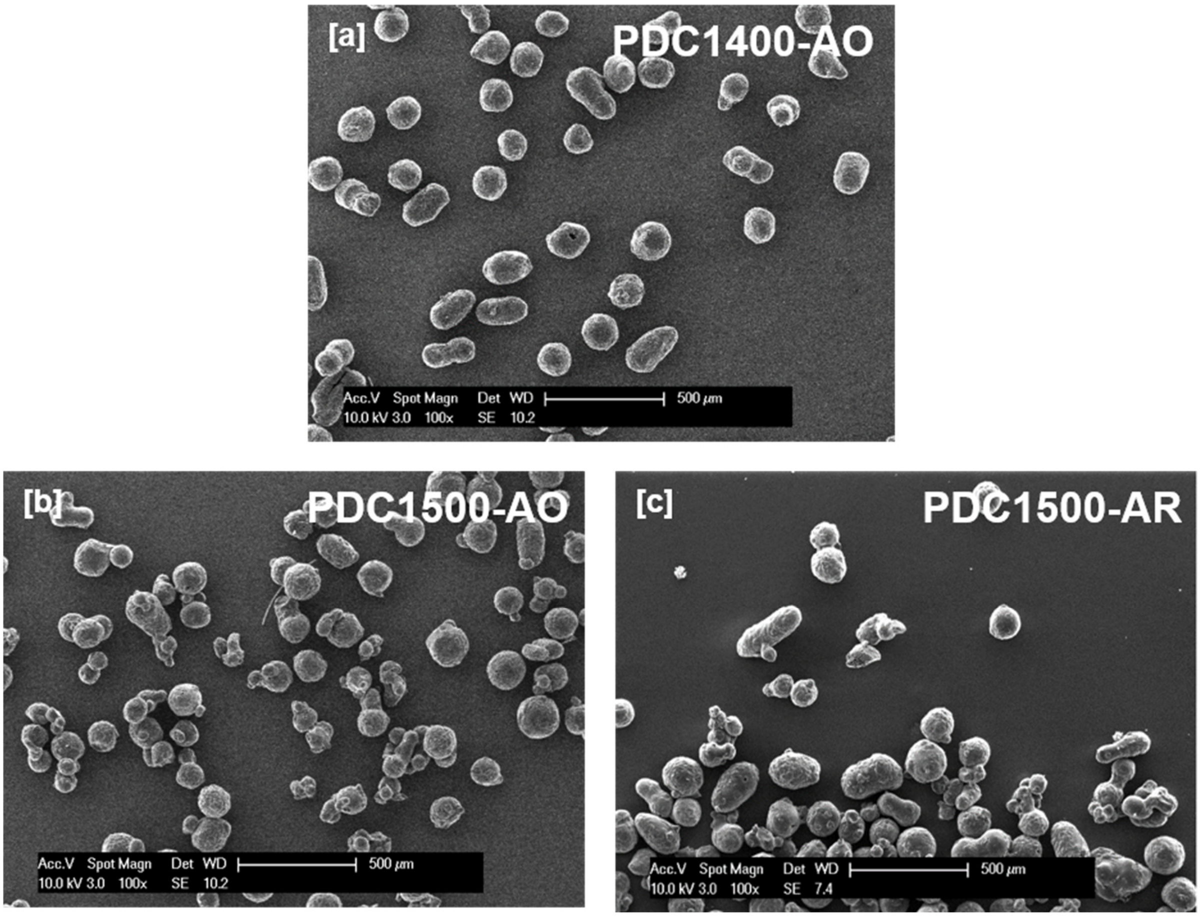

Figure S15: SEM pictures of PDC1400-AO [a], PDC1500-AO [b] and PDC1500-AR [c].

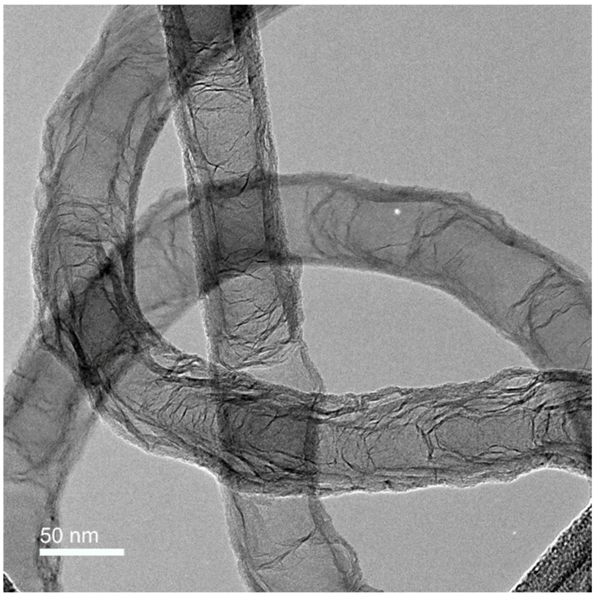

Figure S16: TEM micrograph of the CNT benchmark catalyst.

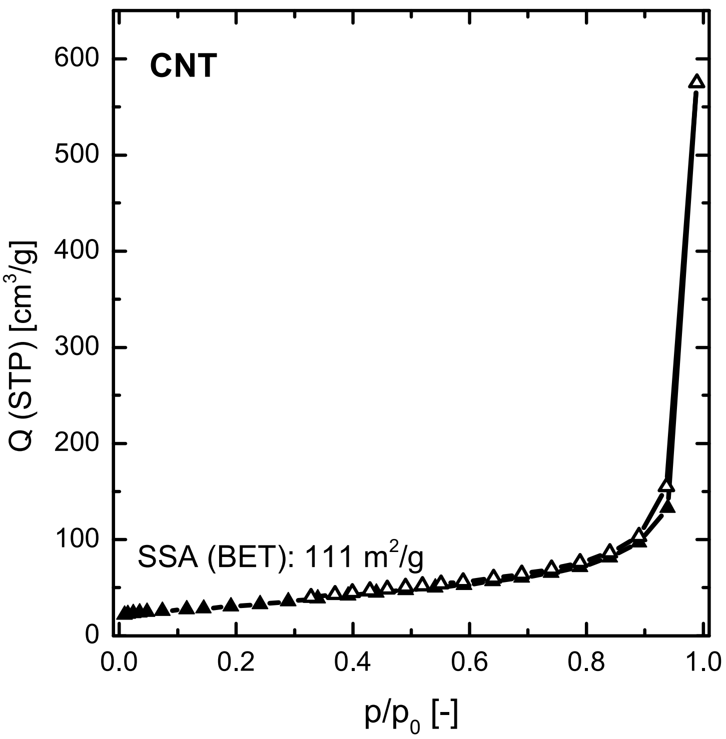

Figure S17: N_2 physisorption isotherm at 77 K of the CNT benchmark catalyst.

<u>Side note S1</u>

The mass-based comparison of the catalytic performance of the PDC materials and the CNT benchmark catalyst was carried out in two tubular quartz reactors with different internal diameters (4 mm for testing of the PDC materials, and 8 mm for the testing of the CNT benchmark.) This approach was necessary due to an inappropriate pressure drop when working with 90 mg of CNT in the reactor with 4 mm internal diameter. However, for both reactors the linear flow velocity was varied and the studies were only carried out within the regime of constant reaction rates under exclusion of film diffusion limitations.

For the extraction of kinetic parameters (O_2, EtOH and temperature variation) a smaller amount of the CNT benchmark catalyst was employed, which enabled the utilization of the 4 mm internal diameter reactor with high linear flow velocity.

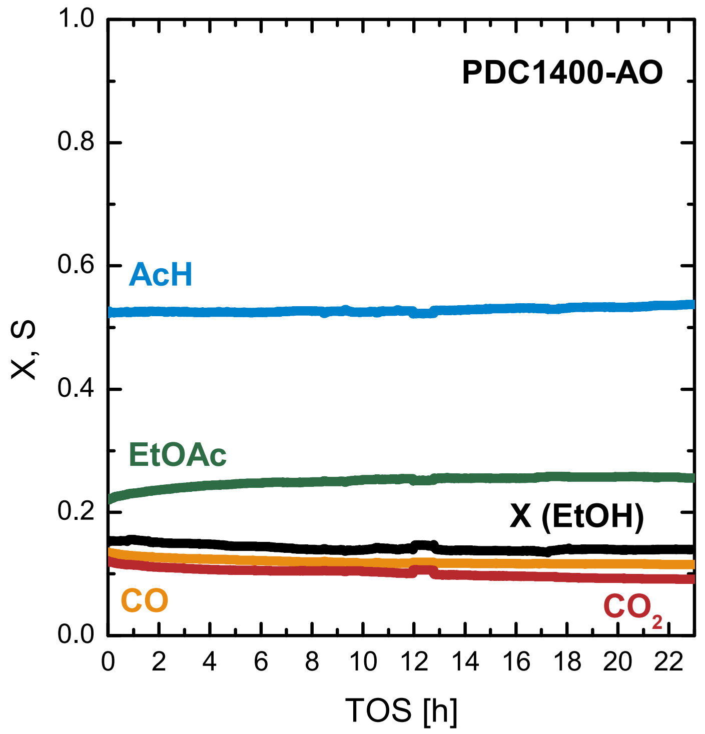

Figure S18: Catalytic performance of PDC1400-AO over 23 h time on stream. Catalytic testing was conducted with 90 mg catalyst at 330 °C in a tubular reactor with 4.3 vol-% EtOH, 10 vol-% O_2 at a total volume flow of 20 mL/min (STP) with He as balance.

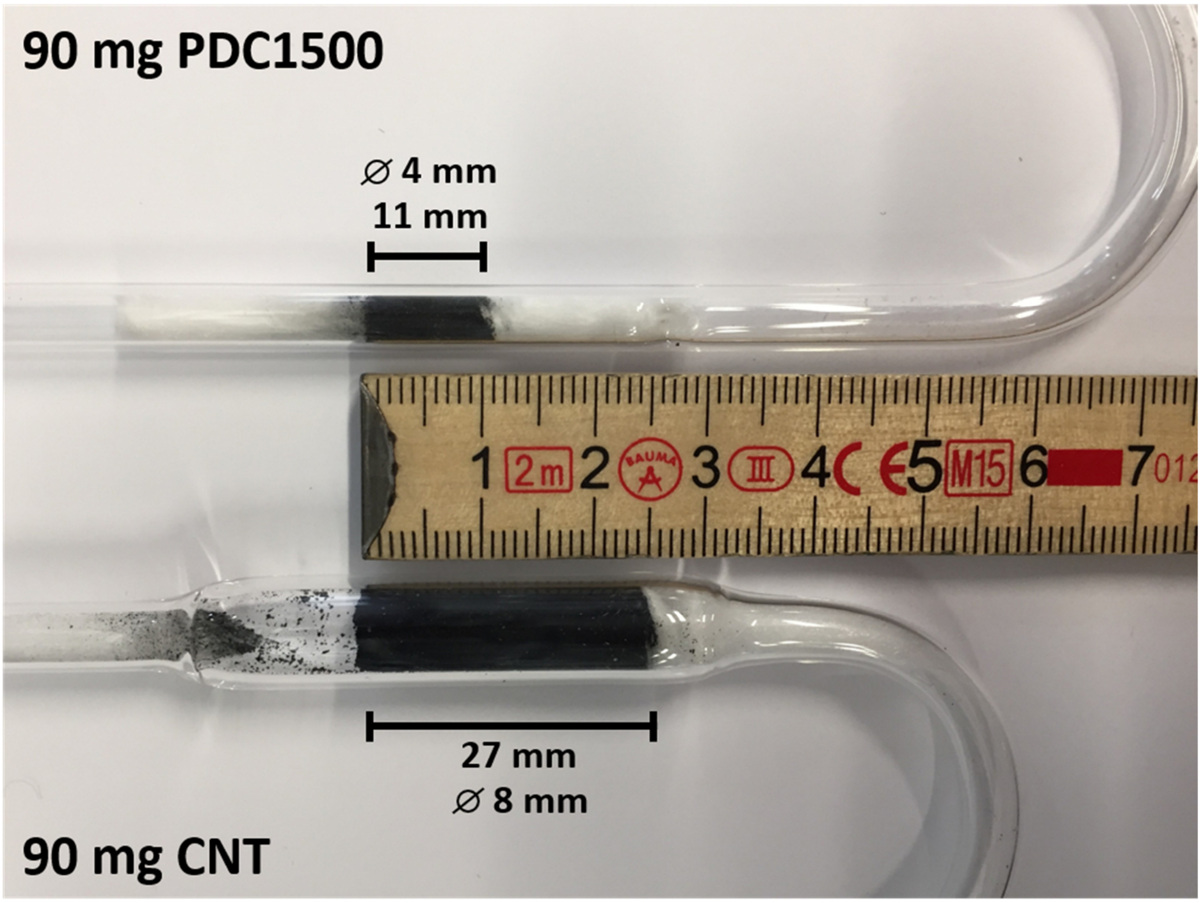

Figure S19: Comparison of catalyst bed volumes for 90 mg of the CNT benchmark and 90 mg of PDC1500-AO.

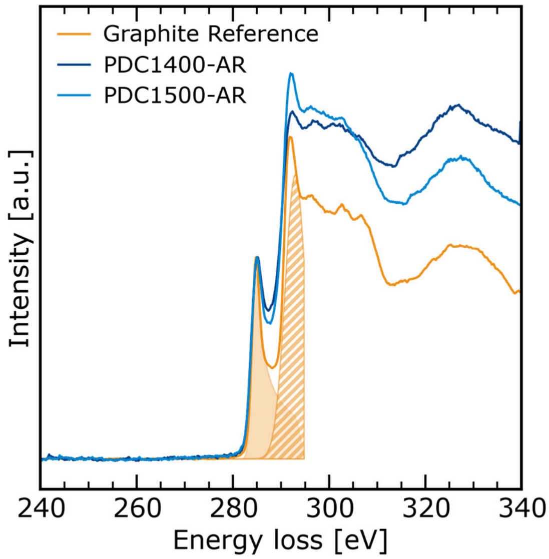

Figure S20: Exemplary EELS spectra of PDC1400-AR and PDC1500-AR compared to a graphite reference.

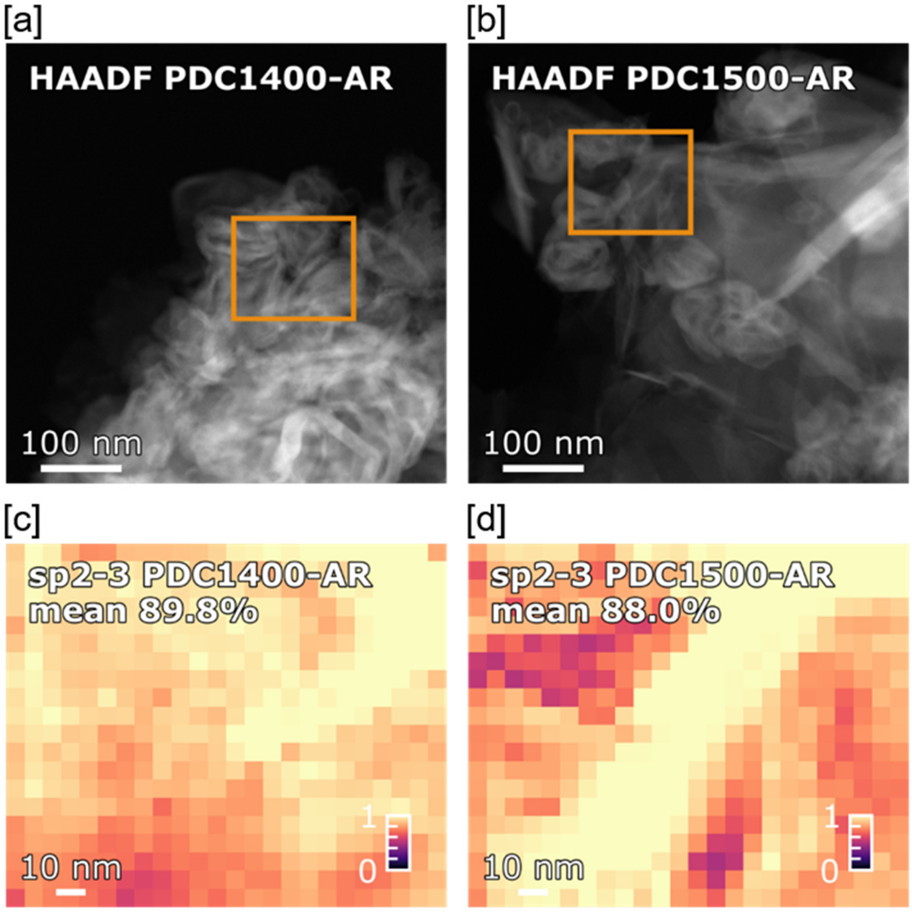

Figure S21: High-angle annular dark-field images of [a] PDC1400-AR and [b] PDC1500-AR. The orange rectangles mark the area in which the EELS mapping was executed. EELS map of [c] PDC1400-AR and [d] PDC1500-AR probing the fraction of sp^2-hybridized carbon of the materials. Bright areas indicate a high sp^2 fraction, dark areas indicate a high sp^3 fraction. All spectra have been background corrected and are normalized for better overview to signal positioned at 285.0 eV.

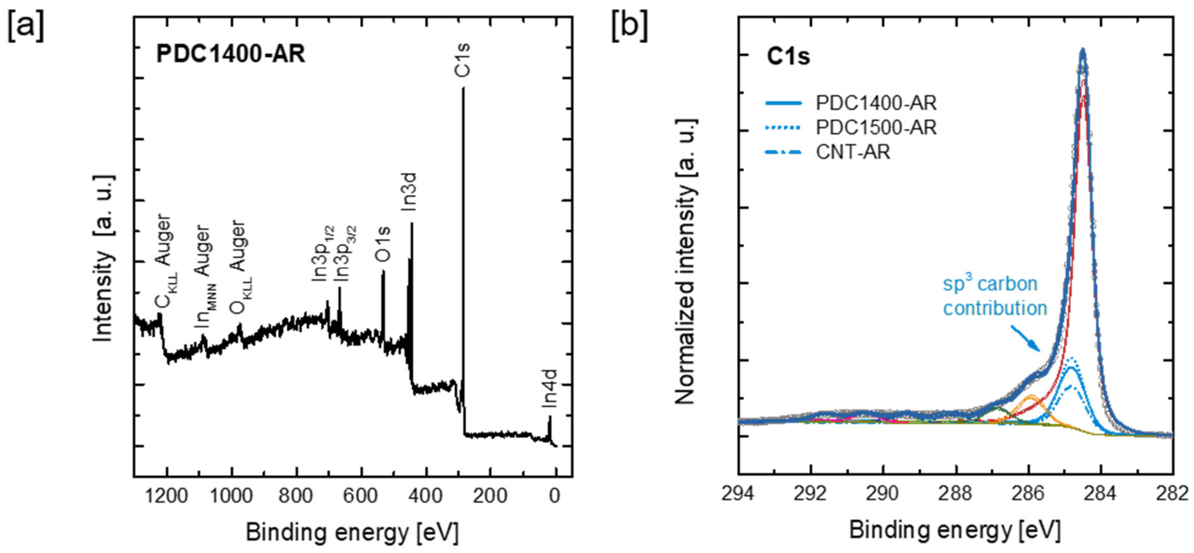

Figure S22: [a] Representative XPS survey scan of PDC1400-AR (for the XPS measurement, the samples were pressed in In foil). [b] Overlay of C1s XP spectra of PDC1400-AR, PDC1500-AR and CNT-AR. The only significant difference was observed in the contribution of sp^3 carbon species, with the CNT benchmark exhibiting a lower sp3 carbon content compared to PDC1400-AR and PDC1500-AR.

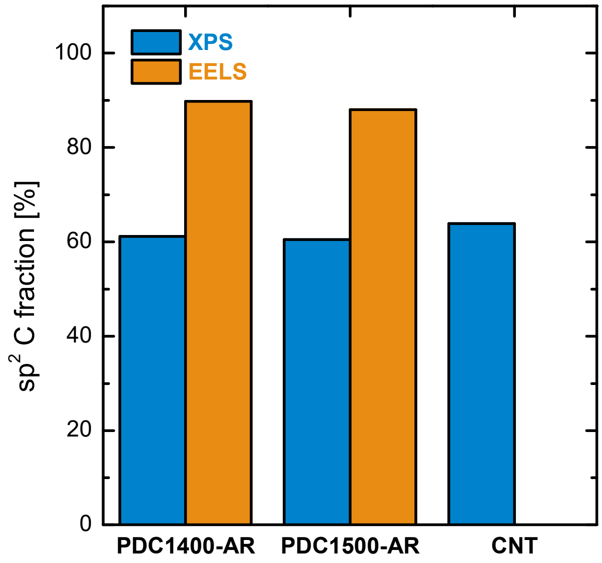

Figure S23: Comparison of the fraction of sp^2 hybridized carbon determined by analysis of the XPS C1s contribution and by averaging the results of the EELS mapping.

Side note S2: Characterization of spent catalysts

N_2-sorption experiments showed in case of PDC1400-AR a slight decrease in micro-/mesoporosity, as the slope of N_2-uptake in the relative pressure region between 0.1 and 0.5 is lower in comparison to PDC1400-AO. While the shape of the hysteresis did not appear to change significantly during reaction, the specific surface area decreases from 640 m^2 g^{-1} to 540 m^2 g^{-1}. In case of PDC1500-AR a small increase in mesoporosity was observed, indicated by a higher N_2-uptake at high relative pressures $0.5<p/p_0<1$. Compared to PDC1500-AO, the isotherm of PDC1500-AR remained otherwise unchanged, while the specific surface area increased slightly from 182 m^2 g^{-1} prior the reaction to 209 m^2 g^{-1} after the reaction (Figure 2). Raman spectroscopy of the used catalysts revealed in case of PDC1400-AR a further decrease of the average I_D/I_G ratio from 0.91 to 0.82, while the distribution of I_D/I_G values further narrowed. For PDC1500-AR, the opposite trend was observed: the average I_D/I_G ratio slightly increased from 0.39 for the PDC1500-AO to 0.42 for PDC1500-AR, while the distribution broadened (Figure S6 – S9). TPO showed for PDC1400-AR, the ratio of carbon of low oxidation resistance to carbon of high oxidation resistance decreases slightly in comparison to PDC1400-AO (LOR:HOR 63:37). In case of PDC1500-AR, only one carbon species remains after subjection to catalysis, which is represented by a comparably broad peak of the derivation of the mass loss curve. In case of PDC1400-AR as well as PDC1500-AR, XRD shows no significant differences compared to PDC1400-AO and PDC1500-AO, respectively (Figure 2c). TEM analysis of PDC1500-AR indicated no apparent difference in comparison with PDC1500-AO (Figure 1d, Figure S14). The state of hybridization (fraction of sp^2 hybridized carbon of the materials), probed by XPS and EELS for the spent catalysts shows no significant differences between PDC1400-AR and PDC1500-AR (Figures S20-23). In case of XPS a surface concentration of sp^2 hybridized carbon of 61 at-% was determined for PDC1400-AR, while PDC1500-AR exhibited 60 at-%. Similarly, the average sp^2 fraction determined by EELS

mapping did not produce significant differences, yielding an sp^2 content of 90 % for PDC1400-AR and 88 % for PDC1400-AR.

Table S2: Comparison of physico-chemical and catalytic properties of PDC1400-AR, PDC1500-AR and the benchmark CNT catalyst.

	**PDC1400-AR**	**PDC1500-AR**	**CNT**
**Specific surface area [m^2 g^{-1}]**	540	209	111
**Metal content [wt-%]**	-	0.041 ± 0.001	-
**Bulk density [g cm^{-3}]**	0.65	0.65	0.07
**Surface oxygen content [at-%]***	7.8	7.8	8.9
**Raman I_D/I_G**	0.83	0.42	-
**EtOH conversion [%]**	14	32	38
**AcH selectivity [%]**	54	82	79
**STY [kg_{AcH} m^{-3} h^{-1}]**	49	171	19

*Determined by XPS.

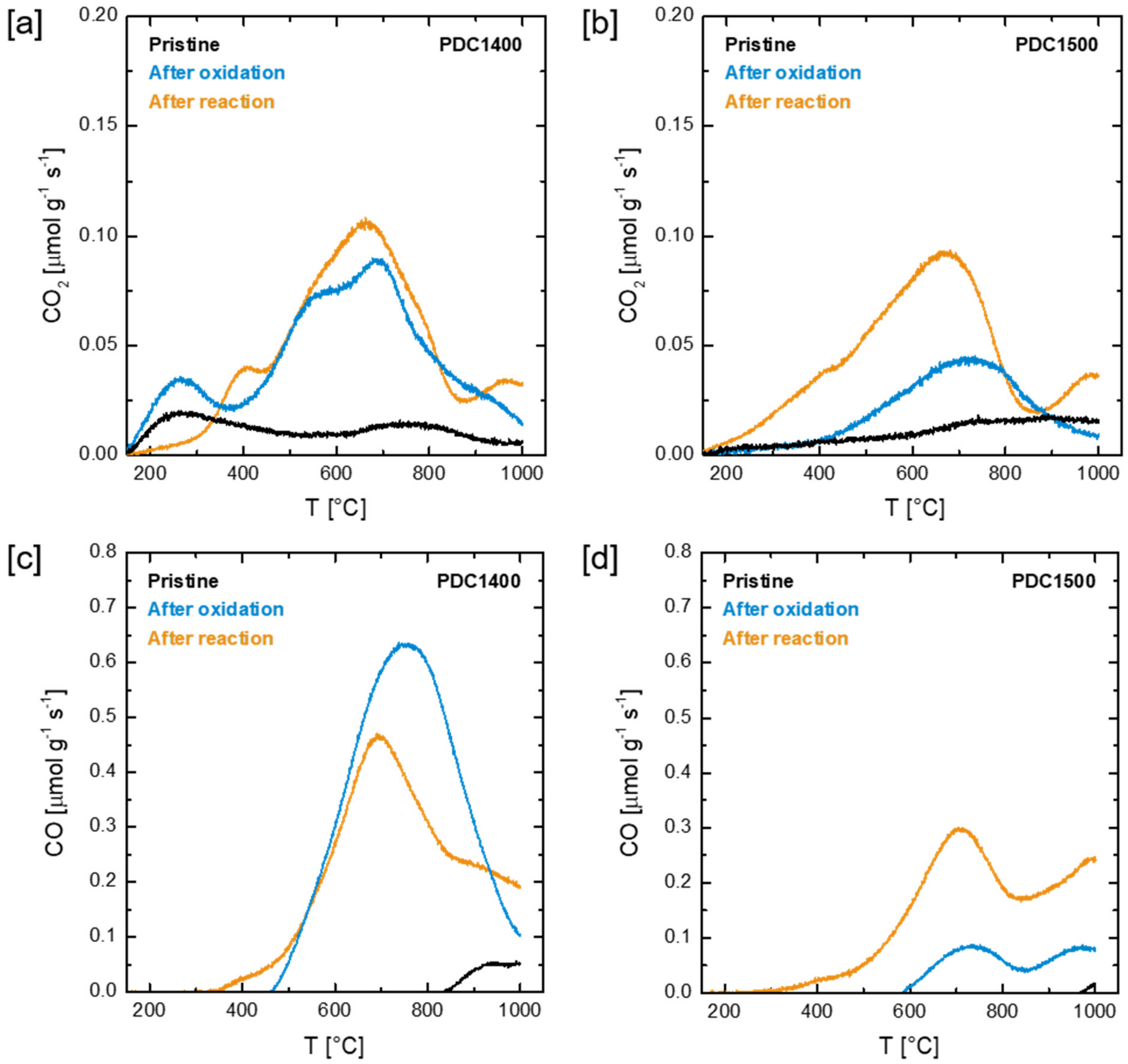

Figure S24: TPD CO_2 emission profiles of [a] PDC1400 and [b] PDC1500 materials. TPD CO emission profiles of [a] PDC1400 and [b] PDC1500 materials.

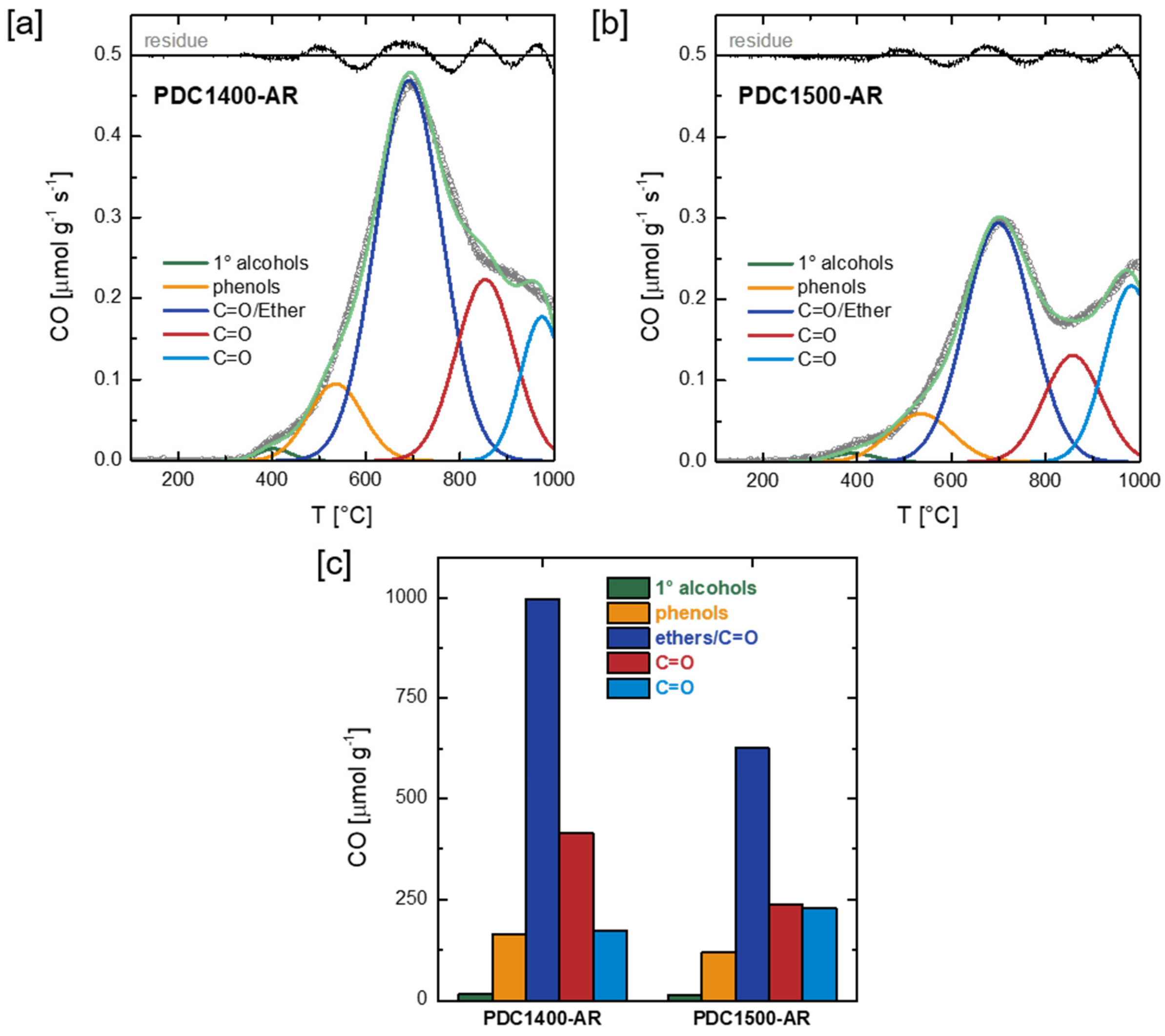

Figure S25: Fitting of the TPD CO emission profiles as proposed by FIGUEIREDO, PEREIRA and co-workers.[4] Analysis of the TPD CO emission profile of [a] PDC1400-AR, [b] PDC1500-AR and [c] the comparison of the extracted concentrations of CO emitting surface species on PDC1400-AR and PDC1500-AR.

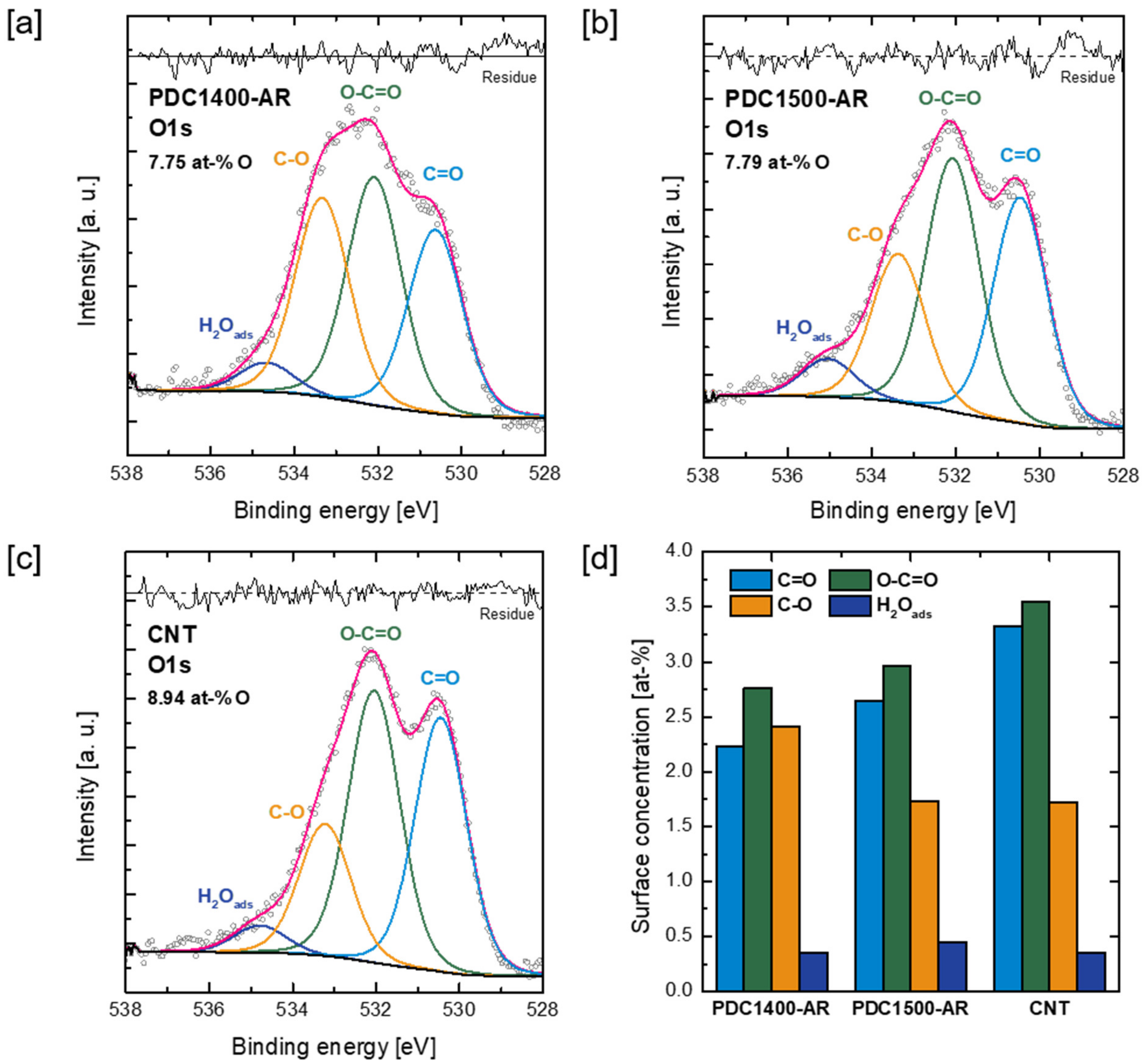

Figure S26: Analysis of the XPS O1s region of [a] PDC1400-AR, [b] PDC1500-AR and [c] the CNT benchmark after the reaction. [d] Comparison of the extracted surface concentrations of C=O species (quinones, ketones) O-C=O species (carboxylic acid derivates) and C-O species (alcohols, ethers) occupying PDC1400-AR, PDC1500-AR and the CNT benchmark catalyst after the reaction.

Side note S3: Impact of carbon backbone of PDC1400 and PDC1500 on catalytic activity

In terms of TEM, TPO and Raman spectroscopy, no obvious difference with apparent impact on the catalytic behaviour between both catalysts after the reaction (PDC1400-AR and PDC1500-AR) could be determined (Figures 1, 2b and Figures S7-9 and S13-14). Both materials exhibit similar microstructures, with PDC1400-AR exhibiting a higher mass fraction of amorphous carbon.

Looking at the hybridization of the carbon in both materials, XPS as well as EELS characterization found very similar fractions of sp^2 hybridized carbon atoms (Figures S20-23). As can be seen from EELS mapping, both materials exhibit a heterogeneous microstructure in terms of hybridization, which reflects the disordered structures observed by TEM analysis.

Concerning N_2-physisorption, however, there was a significant difference between both materials, but in favour of PDC1400-AR, as it exhibits a much higher specific surface area compared to PDC1500-AR (540 m^2 g^{-1} vs. 209 m^2 g^{-1}, Figure 2a). In this context, the specific surface area does not seem to be of major importance for the catalytic performance, as PDC1500-AR provided much higher EtOH conversions and AcH selectivities.

Apart from the non-significant differences in specific surface area, differences could be observed in the long range order of graphitic domains of PDC1500 and PDC1400. Specifically, XRD analysis provided evidence for the presence of a graphitic phase of high stacking order (stacking of graphene layers) in PDC1500 or better of a stacking distance closer to ideal graphite, which was absence in PDC1400 (Figures 2c and d, Figure S11). In addition to differences in long range order, TPD and XPS provide evidence of a higher concentration of potential active species in case of PDC1500-AR compared to PDC1400-AR (Figures S24-26).

Regarding the analysis of the TPD CO emission profile of PDC1400-AR and PDC1500-AR it can be noted, that the overall emission of CO is much higher for PDC1400-AR (1766 μmol g^{-1})

than for PDC1500-AR (1227 µmol g^{-1}, Figure S25). This observation provides evidence for the presence of a high number of bystander species, as this high number of CO emitting surface oxides does not translate into catalytic activity. However, there is one high-temperature stable species with an CO emission maximum of ~980 °C that is associated with carbonyl species such as quinones among others, which is present in higher concentration on PDC1500-AR (229 µmol g^{-1} for PDC1500-AR vs. 173 µmol g^{-1} for PDC1400-AR).[4] It is possible that this species, in combination with phenols, represents the active redox pair in EtOH oxidation reactions.

Analysis of the XPS O1s region shows in case for PDC1500-AR a higher contribution of an oxygen species which exhibits a binding energy around 530 eV compared to PDC1400-AR (2.2 at-% for PDC1400-AR vs. 2.6 at-% for PDC1500-AR, Figure S26). The region of this binding energy is, similar to the results of the analysis of the TPD CO emission profile, associated with the presence of surface ketones and quinones.[6] However, the differences in the surface oxide profiles between PDC1400-AR and PDC1500-AR are by far not as significant as the difference in catalytic activity and selectivity, and hint at a rather large concentration of bystander species that do not contribute to the catalytic activity of the carbon material.

In summary, the only differences that speak for PDC1500-AR as the “better” dehydrogenation catalyst are found to be the presence of a graphitic phase with a close-to-ideal stacking distance of the graphite layers and small deviations in the surface oxide profile, which suggest a slightly higher concentration of potential active sites on PDC1500-AR in comparison to PDC1400-AR. In this context, our hypothesis, that the “carbon backbone” of a given surface oxide plays a crucial role in the redox activity of this particular functional group, has been developed.

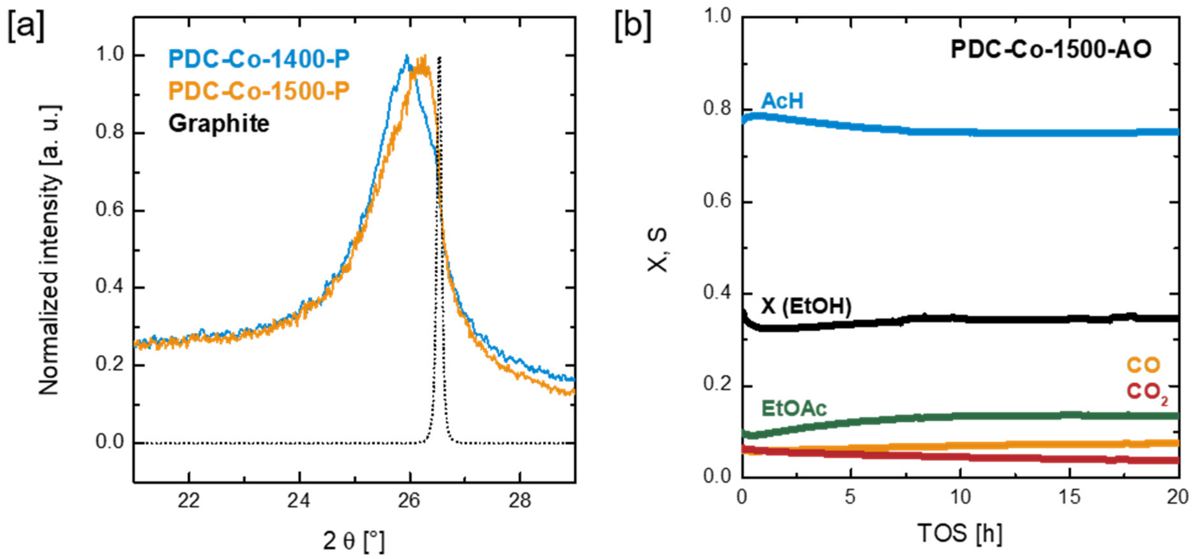

Figure S27: [a] Comparison of the (002) reflection in X-ray powder diffraction patterns of polymer derived carbons graphitized with Co at 1400 and 1500 °C with a graphite reference. [b] Catalytic performance of the PDC graphitized with Co at 1500 °C in the oxidation of ethanol towards acetaldehyde over 20 h time on stream. Catalytic testing was conducted using 90 mg of catalyst (after acid leaching and preoxidation) at 330 °C in a tubular fixed bed reactor with 4.3 vol-% EtOH, 10 vol-% O_2 at a total volume flow of 20 mL/min (STP) with He as balance.

Side note S4: WEISZ-PRATER criterium for the evaluation of diffusion limitation

The WEISZ-PRATER criterion was calculated for the consumption of EtOH on PDC at 330 °C using the following set of equations.

$$\Psi = l_c^2 \cdot \frac{n+1}{2} \cdot \frac{r_{obs} \cdot \rho_{Kat}}{D_{eff} \cdot c_0} = 3.6 \cdot 10^{-5} \qquad \text{eq. S1}$$

$$l_c = \frac{1}{3} r_{Cat} \qquad \text{eq. S2}$$

$$D_{eff} = D_{He,EtoH} \cdot \frac{\varepsilon}{\tau} \qquad \text{eq. S3}$$

Table S3: Calculated and estimated constants for the determination of the WEISZ-PRATER criterion.

Abbrevation	Meaning	Value
l_c	Characteristic length of a catalyst particle	$1.67 \cdot 10^{-5}$ m
r_{Cat}	Radius of a catalyst particle	$5 \cdot 10^{-5}$ m
n	Reaction order of EtOH consumption	0.59
r_{obs}	Observed rate of EtOH consumption	$1.66 \cdot 10^{-3}$ mol kg^{-1} s^{-1}
ρ_{Cat}	Density of a catalyst particle	1000 kg m^{-3}
D_{eff}	Effective diffusion coefficient	$6.05 \cdot 10^{-6}$ m^2 s^{-1}
$D_{He,EtOH}$	Diffusion coefficient of EtOH in He at 330 °C (estimation according to HIRSCHFELDER *et al.*[7])	$1.513 \cdot 10^{-4}$ m^2 s^{-1}
c_0	EtOH feed concentration	1.676 mol m^{-3}
ε	Effective porosity	0.3
τ	Tortuosity factor	7.5

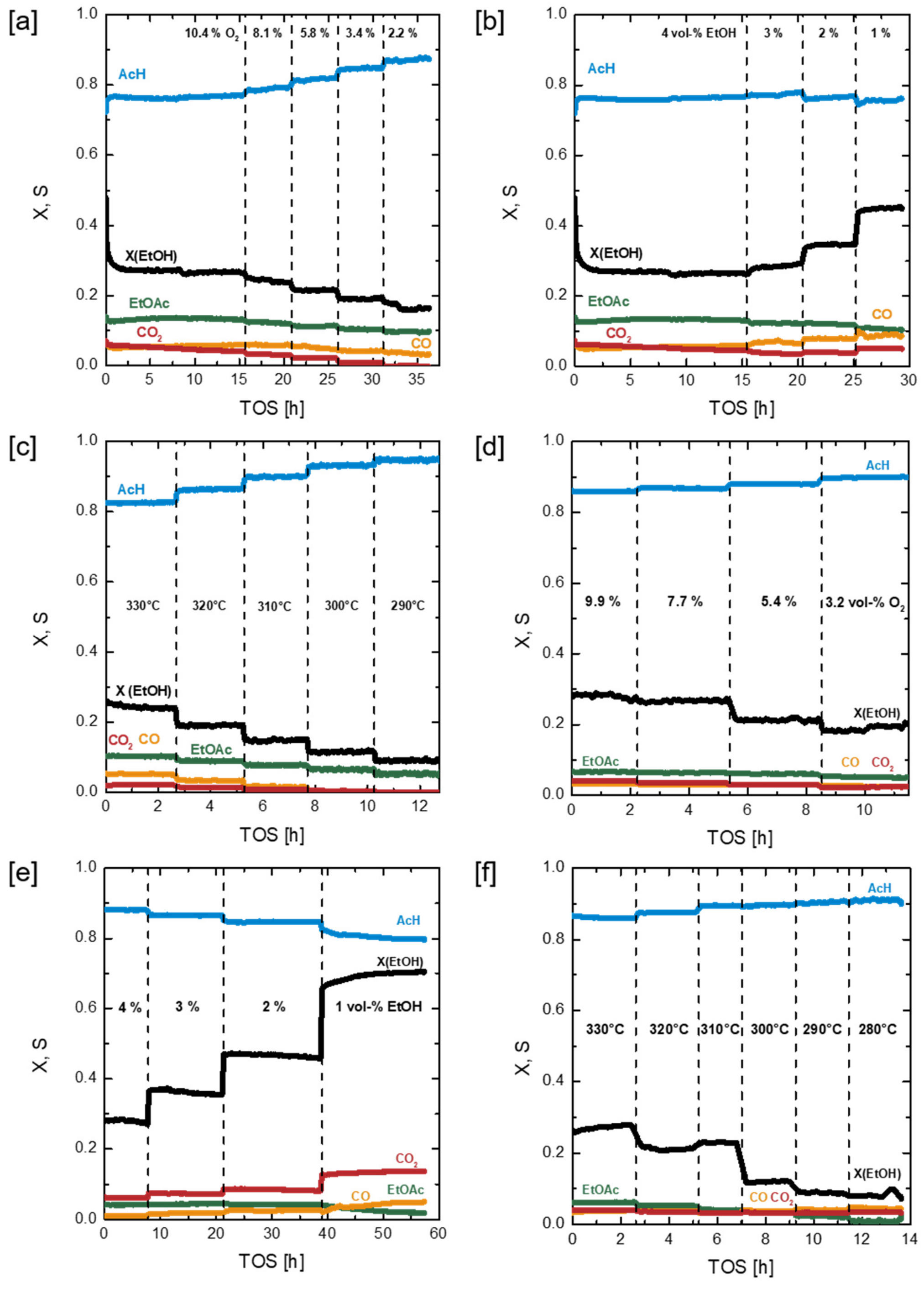

Figure S28: Variation of the oxygen concentration [a], ethanol concentration [b] and temperature [c] for PDC1500-AO. Variation of the oxygen concentration [d], ethanol concentration [e] and temperature [f] for the CNT benchmark catalyst. If not stated otherwise, 4.3 vol-% EtOH, 10 vol-% O_2 were employed at 330 °C at a flow rate of 20 mL min^{-1} (He as balance).

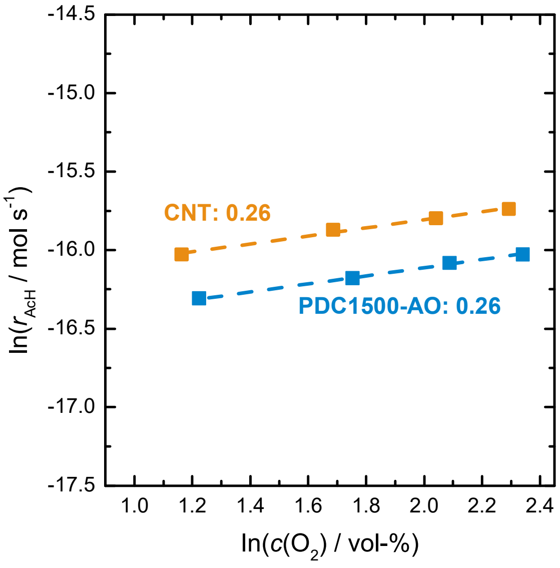

Figure S29: Dependence of the acetaldehyde formation rate on the oxygen concentration: Determination of the reaction order.

REFERENCES

[1] S.-H. Chai, J. Y. Howe, X. Wang, M. Kidder, V. Schwartz, M. L. Golden, S. H. Overbury, S. Dai, D.-e. Jiang, *Carbon* **2012**, *50*, 1574.

[2] P. Mallet-Ladeira, P. Puech, C. Toulouse, M. Cazayous, N. Ratel-Ramond, P. Weisbecker, G. L. Vignoles, M. Monthioux, *Carbon* **2014**, *80*, 629.

[3] N. Bernier, F. Bocquet, A. Allouche, W. Saikaly, C. Brosset, J. Thibault, A. Charaï, *J. Electron Spectrosc. Relat. Phenom.* **2008**, *164*, 34.

[4] a) J. Figueiredo, M. Pereira, M. Freitas, J. Órfão *Carbon* **1999**, *37*, 1379; b) J.L. Figueiredo, M.F.R. Pereira, M.M.A. Freitas, J.J.M. Órfão *Ind. Eng. Chem. Res.* **2007** *46*, 4110; c) F. Herold, O. Leubner, P. Pfeifer, D. Zakgeym, A. Drochner, W. Qi, B.J.M. Etzold *Carbon* **2021**, *171*, 658.

[5] X. Wang, C. Liang, S. Dai, *Langmuir* **2008**, *24*, 7500.

[6] K. Friedel Ortega, R. Arrigo, B. Frank, R. Schlögl, A. Trunschke *Chem. Mater.* **2016**, *19*, 6826.

[7] J. C. Hirschfelder, C. F. Curtiss, R. B. Bird *Molecular Theory of Gases and Liquids* Wiley, New York, **1954**.
